# ﻿New records for the Western Balkans cranefly fauna (Diptera, Tipuloidea) with the description of a new *Baeoura* Alexander (Diptera, Limoniidae)

**DOI:** 10.3897/zookeys.1157.98997

**Published:** 2023-03-31

**Authors:** Levente-Péter Kolcsár, Micha Camiel d’Oliveira, Wolfram Graf, Clovis Quindroit, Kozo Watanabe, Marija Ivković

**Affiliations:** 1 Center for Marine Environmental Studies, Ehime University, Matsuyama, Ehime 790-8577, Japan Ehime University Matsuyama Japan; 2 Naturalis Biodiversity Center, Darwinweg 2, 2333, CR Leiden, Netherlands Naturalis Biodiversity Center Leiden Netherlands; 3 Institute of Hydrobiology and Aquatic Ecosystem Management, University of Natural Resources and Life Sciences Vienna, Vienna, Austria University of Natural Resources and Life Sciences Vienna Vienna Austria; 4 Groupe d’études des Invertébrés Armoricains, Angers, France Groupe d’études des Invertébrés Armoricains Angers France; 5 Division of Zoology, Department of Biology, Faculty of Science, University of Zagreb, Rooseveltov trg 6, 10000, Zagreb, Croatia University of Zagreb Zagreb Croatia

**Keywords:** New records, Pediciidae, taxonomy, terminalia, Tipulidae, wing

## Abstract

The cranefly (Tipuloidea) fauna of the Western Balkans is still poorly known. In this study, occurrence data of 77 species is reported, of which two species are newly recorded for Albania, eight species for Bosnia and Herzegovina, twelve for Croatia, and seven for Slovenia, respectively. A new species, *Baeouraneretvaensis* Kolcsár & d’Oliveira, **sp. nov.** is described from Bosnia and Herzegovina, Montenegro, and Slovenia. Images of the habitus, wing, and male and female terminalia of the new species are provided. Furthermore, images of male terminalia and wings of thirteen additional species are presented.

## ﻿Introduction

Craneflies (Tipuloidea) are one of the most species-rich Dipteran group both in Europe and worldwide and represent almost 10% of all known Diptera species worldwide (Evenhuis and Pape 2022; Oosterbroek 2023). At present, 1267 cranefly species belonging to four families (Cylindrotomidae, Limoniidae, Pediciidae, Tipulidae) are reported from Europe (Oosterbroek 2023). In the last decade, our knowledge of the distribution of European craneflies has increased, but nonetheless craneflies are still considered a poorly known insect group. Apart from a few better-studied countries, like the Czech Republic, Finland, or the United Kingdom, the fauna of many countries is still poorly investigated and it is relatively easy to find new or unreported cranefly species (Kolcsár et al. 2021, 2023).

The Western Balkans (Albania, Bosnia and Herzegovina, Croatia, Kosovo, Montenegro, North Macedonia, Slovenia, and Serbia) and within it the Dinaric Mountains are a biodiversity hotspot, especially regarding freshwater and underground taxa (Gaston and David 1994; Bănărescu 2004; Griffiths et al. 2004; Sket et al. 2004; Ivković and Plant 2015). The Dinaric Mountains are the longest uninterrupted karst area in Europe (Mihevc et al. 2010) with one of the most complex hydrological systems. The rivers and streams of Western Balkans are mostly in good or excellent condition and therefore the nickname of this area is “the Blue Heart of Europe” (https://www.balkanrivers.net/en). The Western Balkans are still unexplored and understudied (Kolcsár et al. 2023), especially when it comes to aquatic insect fauna. Therefore, there are many new described species in the last decade, especially regarding Diptera (e.g., Ivković et al. 2012; Kvifte et al. 2013; Pont and Ivković 2013; Andersen et al. 2016; Kvifte and Ivković 2018). For craneflies, the following publications have dealt with the Western Balkans fauna in the last decade: Bilalli et al. (2021), de Jong et al. (2021), Kolcsár et al. (2015a, b, 2017a, b, 2018a, b, 2023), Keresztes et al. (2018a, b), and Starý (2012).

In this paper, we present new records of Limoniidae, Pediciidae, and Tipulidae from the Western Balkans, and we describe a new *Baeoura* Alexander (Limoniidae) species collected along streams and rivers in Bosnia and Herzegovina, Montenegro, and Slovenia. We also present wing and terminalia photos of several species newly recorded from Croatia, Bosnia and Herzegovina, and Montenegro. All the specimens from the Plitvice Lakes were collected as a part of the project “Phenology of aquatic insects” at the National Park Plitvice Lakes, Croatia. Specimens collected from the Neretva River in Bosnia and Herzegovina for this study were collected during the Neretva Science Week 2022 organized by the Scientists for Balkan Rivers Network in July 2022, which forms part of the Blue Heart of Europe campaign.

## ﻿Materials and methods

The specimens were collected using insect nets, aspirators, light traps, and pyramid emergence traps and are preserved in ethanol. The following format is used for the records collected with the pyramid emergence traps: date referring to the date when traps were emptied after one moth run, trap number is given, e.g., “P1” is pyramid emergence trap number 1 (see Kolcsár et al. 2015a). Description of body coloration based on specimens stored in ethanol. The genital structures were studied using a Zeiss Stemi 508 stereomicroscope and an Olympus CX33 microscope equipped with Canon Kiss M digital camera. Layer photos were combined using Zerene Stacker software. General morphological terminology in this study follows Cumming and Wood (2017) and Ribeiro (2008) in case of male terminalia.

Specimens from the following depositories were examined:

**CKLP** Private collection of L.-P. Kolcsár;

**PCCQ** Private Collection of C. Quindroit, Angers, France;

**PCJS** Private Collection of J. Starý, Olomouc, Czechia;

**PCMCO** Private Collection of M.C. d’Oliveira, Haarlem, The Netherlands;

**SMOC** Silesian Museum, Opava, Czech Republic;

**UZC**Collection of M. Ivković at University of Zagreb.

## ﻿Results

### ﻿Taxonomic treatment

#### ﻿Genus Baeoura

Taxon classificationAnimaliaDipteraLimoniidae

Alexander, 1924

3F59F860-A15D-5B79-8D36-D27F38B69240

##### Type species.

*Eriopteranigrolatera* Alexander, 1920 by original designation.

##### Remarks.

*Baeoura* is a species rich genus, including 69 recognized species, prior to this article. The majority of the species is known from the Oriental region (43 species), but the genus also occurs in the Afrotropics (11 species), the Palearctic (4 species in Eastern Palaearctic and 10 species in Western Palaearctic) and one species from the Neotropics (Oosterbroek 2023). Recently two new species were described from the Western Palaearctic, *B.staryi* Driauach & Belqat, 2015 from Morocco and *B.rotherayi* Hancock, 2020 from Spain.

#### ﻿ Baeoura neretvaensis

Taxon classificationAnimaliaDipteraLimoniidae

Kolcsár & d’Oliveira sp. nov.

307DD2D1-0778-5B27-934E-8634573C31B2

https://zoobank.org/F5064F12-97F3-4B8D-AABF-7B02DE749710

**Figure 1.**
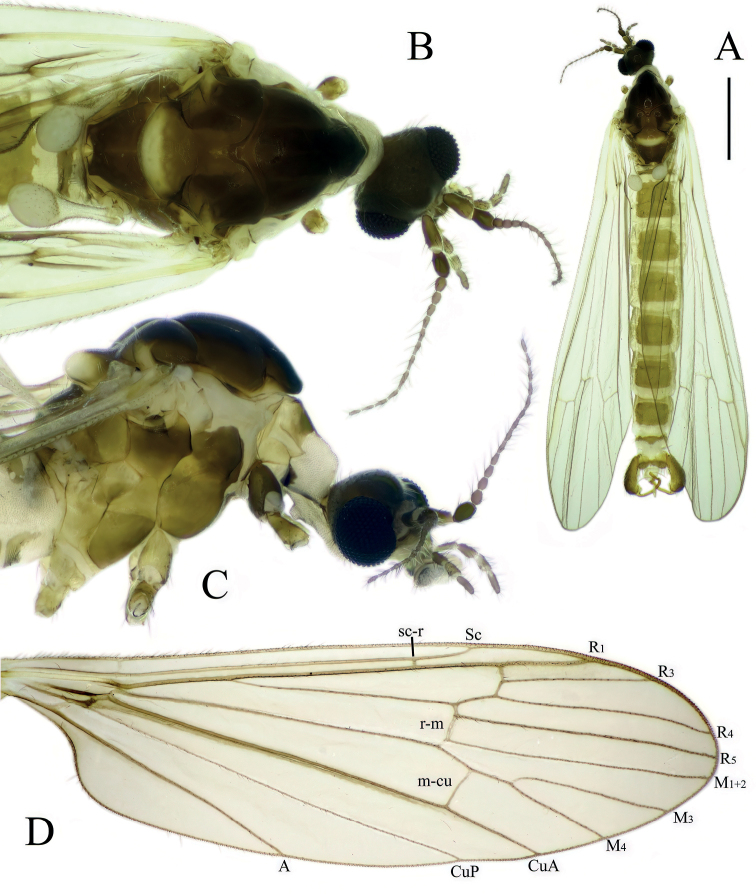
*Baeouraneretvaensis* sp. nov. Paratype: Ulog, Neretva at Ulog Camp site (SMOC) **A** habitus of male **B** head and thorax, dorsal view **C** head and thorax, ventral view **D** wing. Scale bar: 1 mm.

**Figure 2.**
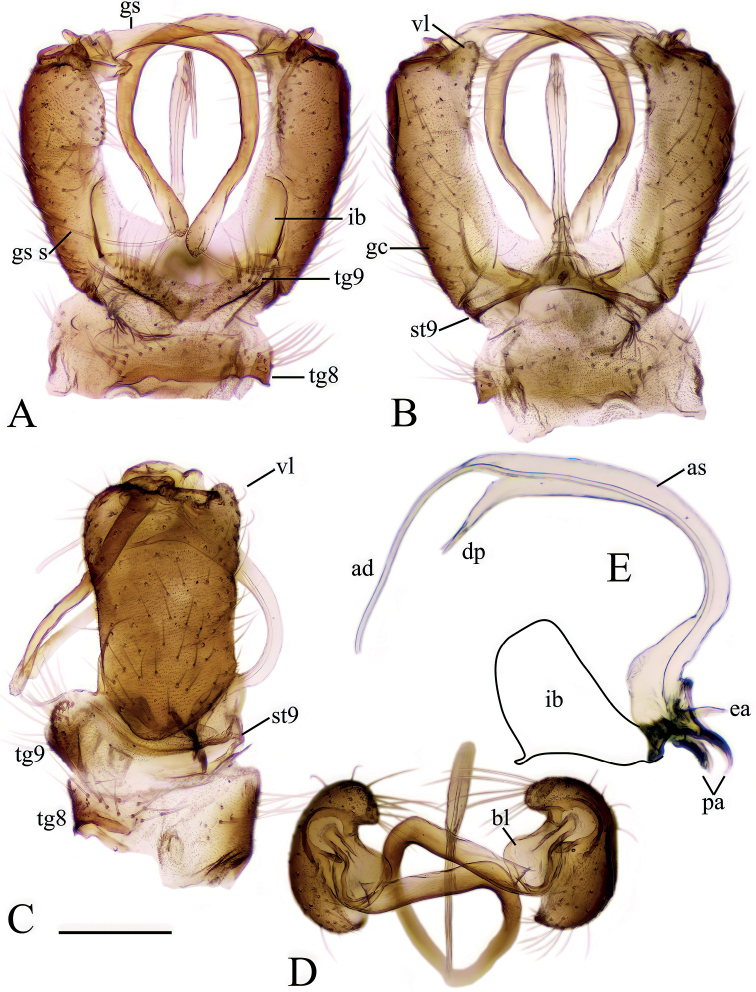
Male terminalia of *Baeouraneretvaensis* sp. nov. Paratype: Ulog, Ulog on Neretva River (SMOC) **A** dorsal view **B** ventral view **C** lateral view **D** caudal view **E** aedeagal complex, lateral view (interbase broken and illustrated with outline). Scale bar: 0.25 mm. Abbreviations: **ea** – ejaculatory apodeme; **ad** – aedeagus; **as** – aedeagal sheath; **bl** – basal lobe of gonostylus; **dp** – dorsal process of aedeagal sheath; **gc** – gonocoxite; **gs** – gonostylus; **gs s** – gonostylar seta; **ib** – interbase; **pa** – paramere; **st** – sternite; **tg** – tergite; **vl** – ventral lobe of gonostylus.

**Figure 3.**
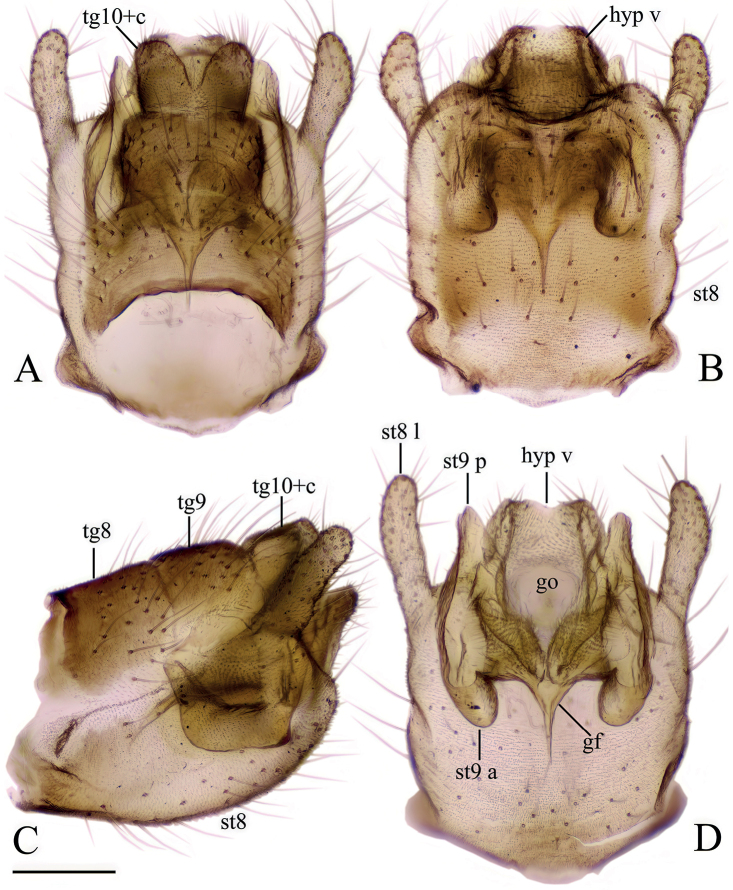
Female terminalia of *Baeouraneretvaensis* sp. nov. Paratype: Ulog, Ulog on Neretva River (SMOC) **A** dorsal view **B** ventral view **C** lateral view **D** sternite 8, hypogynial valvae, sternite 9 and genital fork, inner view. Scale bar: 0.2 mm. Abbr.: **hyp v** – hypogynial valve; **gf** – genital fork; **go** – genital opening; **st** – sternite; **st8 l** – lobe of sternite 8; **st9 a** – sternite 9 anterior invagination; **st9 p** – sternite 9 posterior lobe; **tg** – tergite, **tg10+c** – fused segment of tergite 10 and cerci.

**Figure 4.**
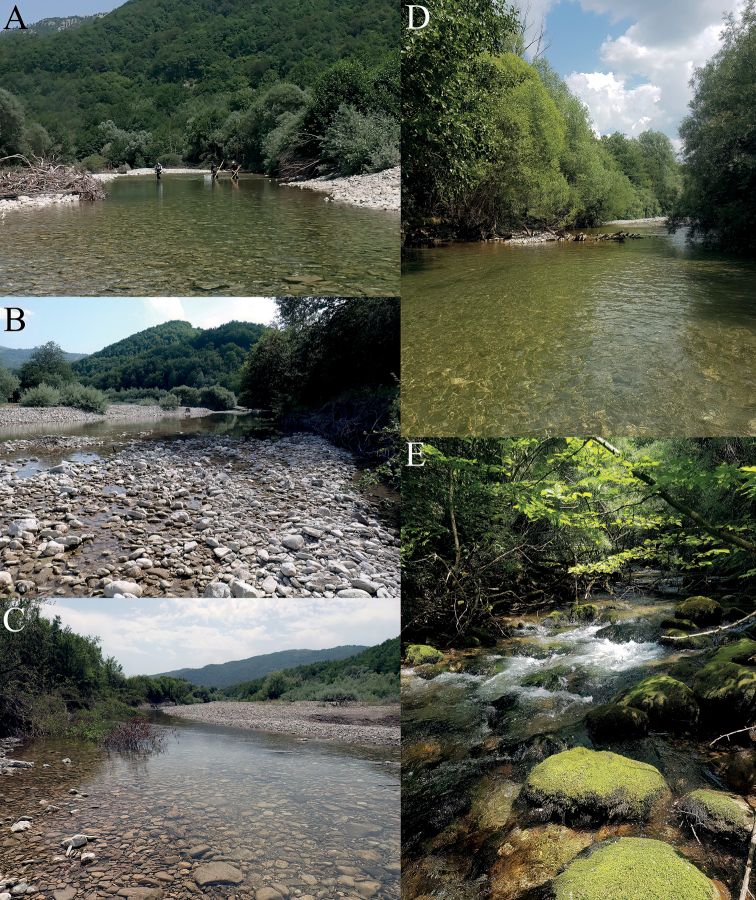
Neretva River and confluence **A–C** ulog on Neretva River; 43.42414°N, 18.30837°E**D** Cerova on Neretva, 43.37887°N, 18.35621°E**E** Krupac, confluence to the Neretva River, 43.32942°N, 18.42574°E. Photographs: Wolfram Graf (**A–C**), Marija Ivković (**D, E**).

**Figure 5.**
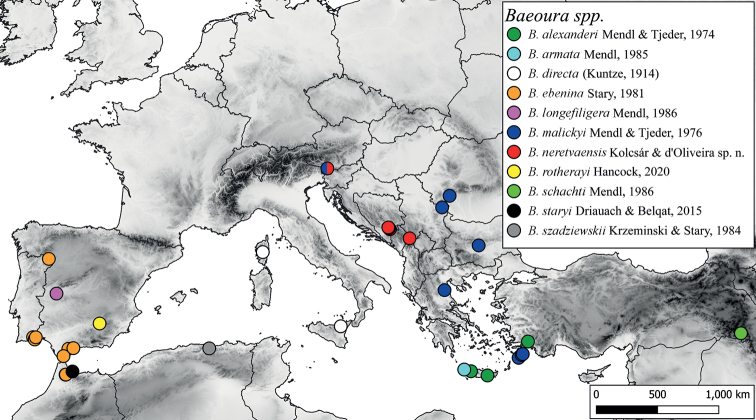
Distribution of *Baeoura* Alexander species in the Western Palearctic.

Figs 1 , 2 , 3 , 4 , 5

##### Type material.

***Holotype*. Bosnia and Herzegovina** • male; Ulog, Neretva River at Ulog Camp site; alt. 650 m; 43.41714°N, 18.31205°E; 28 Jun. 2022; W. Graf leg.; HOLOTYPE *Baeouraneretvaensis* Kolcsár & d’Oliveira, sp. nov. [red label]; SMOC.

***Paratypes*. Bosnia and Herzegovina** • 3 females; Ulog, Neretva at Ulog Camp site; 43.41714°N, 18.31205°E; alt. 650 m; 28 June 2022; leg. W. Graf; SMOC • 2 males, 2 females; Krupac, Krupac Confluence to Neretva River; 43.32942°N, 18.42574°E; alt. 775 m; 29 June 2022; leg. M. Ivković; 1 male in UZC, 1 male and 2 females in SMOC • 1 female; Cerova, Cerova on Neretva; 43.37887°N, 18.35621°E; alt. 695 m; 30 June 2022; leg. M. Ivković; SMOC • 6 males, 13 females; Ulog, Ulog on Neretva River; 43.42414°N, 18.30837°E; alt. 640 m; 29 June 2022; leg. W. Graf; 1 male and 1 female in PCJS; 4 males and 11 females in SMOC, 1 male and 1 female in CKLP. **Montenegro** • 1 female; Berane, on window in town; 42.8436°N, 19.8666°E; alt. 685 m; 07 July 2012; leg. M. Ivković; UZC. **Slovenia** • 2 females; Gorenjska, Juliske alpe, Gozd Martuljek, River Sava; in small woodland on the banks of river Sava; 46.483°N, 13.838°E; alt. 740 m; 20 August 2019; leg. M.C. d’Oliveira; PCMCO.

##### Diagnosis.

General coloration dark brown, with lateral parts of thorax striped. Scutellum posterior margin whitish. Wing without any markings, hyaline. Gonocoxite long and narrow, without prominent dorsal lobe. Gonostylus very long, narrow, and strongly curved, with a long seta at tip and a flat, blade-like lobe at base. Aedeagal sheath large, strongly curved dorsally, laterally flattened, with a forked process at 3/5 of its length from the base. Female terminalia with a pair of finger-like lobes on sternite 8, longer than cercus or hypogynial valve. Genital chamber complex and strongly sclerotized, sternite 9 with a pair of triangular lobes on the posterior edge, and a pair of finger-like anterior invaginations.

##### Description.

**Male.** Body length 5.5–6.5 mm, wing length 4.5–5.5 mm. General color dark brown, with lighter abdomen (Fig. 1A).

***Head*.** Wider than long. Eyes small and dorsally widely separated, ~ 1/2 as wide as narrowest point between eyes (Fig. 1B); eyes also separated from each other ventrally too, ~ 1/2 of width of eye. Eyes small, separated dorsally and ventrally (Fig. 1B). Dorsal separation ~ 1/2 as wide as narrowest point between eyes (Fig. 1B), ventral separation ~ 1/2 of wide of eye. Vertex dark brown. Rostrum short, pale brown to brown. Palpus 5-segmented, uniformly brown, or slightly paler at apex; palpomeres 2–5 similar in size. Antenna ~ 2–2.5× longer than head, reaching beyond prescutum if bent backward (Fig. 1B, C). Scape brown, cylindrical 2–2.5× longer than wide; pedicel dark brown, shorter than scape, slightly enlarged, drop-shaped, 2× wider than first flagellomere. Flagellum 13-segmented, brown, first flagellomere drop-shaped, subsequent flagellomeres gradually decreasing in wide and increasing in length toward apical segment, last flagellomere cylindrical. Basal flagellomeres with 2–4 longest verticils on dorsal and lateral sides, start from flagellomere 5 with five or six verticils; length of verticils subequal to length of corresponding flagellomeres.

***Thorax*.** Dark brown dorsally (Fig. 1B), lateral parts stripped (Fig. 1C), formed by lighter and darker parts. Cervical sclerite black, roughly angular with a long extension connecting to head. Pronotum flat, anterior part brown posterior part pale brown. Prescutum and anterior part of scutum dark brown with four broad, darker, less distinct, longitudinal stripes (probably more visible on dry specimens). Central two stripes are fused anteriorly on prescutum, stripes cease near transverse suture. Outer stripes start on sides of posterior scutum, ceasing directly at transverse suture. Posterior part of scutum brown, with two longitudinal darker patches, lateral corner of scutal lobe distinctly yellowish brown. Scutellum brown anteriorly, posterior margin conspicuously white. Mediotergite dark brown. Proepisternum yellowish, pleuron, and posterior basalare white to yellowish white. Coxa 1, anepisternum, and anepimeron dark brown. Trochanters 1, 2, katepisternum, and meron brown. Coxae 2, 3, trochanter 3, and metaepisternum light brown. Femora brown, slightly darkening towards apex; tibiae and tarsi brown. Wing yellowish tinged, 3–3.2× as long as wide (Fig. 1D). Stigma inconspicuous, whitish subhyaline with backlight. Vein Sc ending between level of forks of Rs and R2+3+4; crossvein sc-r situated on level of 4/5 of Sc (measured from crossvein h); R2+3+4 short, ~ 1/5–1/4 length of R4; R2+3 ~ as long as R2, almost perpendicular; M1+2 slightly longer than R5; crossvein r-m, 2–4× longer than basal section of M1+2; cell dm opened, by atrophy of crossvein m-m; M4 ~ 1/4–1/3× longer than M3+4; cross vein m-cu ca. middle of M3+4, relatively long; wing margin between tips of M1+2 and M3 similar in length as between M3 and M4, and between M4 and CuA, and ca. 1/2 in length than distance between CuP and A, CuP and A almost straight. Halter whitish, ~ 0.5 mm (Fig. 1B).

***Abdomen*.** Tergites and sternites uniformly pale brown; terminalia slightly darker (Fig. 1A).

***Male terminalia*** (Fig. 2). Relatively large and prominent. Tergite 8 very narrow, posterior margin fits over anterior part of tergite 9 (Fig. 2C). Tergite 9 narrow, anterior part weakly sclerotized; posterior part with pair of round lobes, bearing long setae (Fig. 2A). Sternite 9 present as narrow band. Gonocoxite long, ~ 3× longer than wide at middle with short ventral lobe (Fig. 2B, C). Gonostylus narrow and very long, directed dorsally (Fig. 2C) and strongly curved (Fig. 2A, B); basally with a flat plate, whose margin round from posterior view (Fig. 2D); tip of gonostylus slightly widened, with a perpendicular, very long, subhyaline gonostylar seta (Fig. 2A). Aedeagal complex long, as long as gonocoxite (Fig. 2 A, B, C). Interbase flattened, blade-like, widely fused with mesal surface of gonocoxite (inseparable from gonocoxite without breaking it), ~ 1/3 length of gonocoxite (Fig. 2A); tip convex or slightly concave in lateral view (Fig. 2E); both interbases medially fused. Aedeagal sheath strongly curved dorsally, narrowest near base, gradually broadening distally, widest at 3/5 of its length, produced into a very long filament-like aedeagus and a dorsal forked extension (Fig. 2E). Ejaculatory apodeme short, rod-shaped, directed ventrally. Parameres short, slightly curved dorsally in later view (Fig. 2E) and directed laterally in ventral view.

**Female.** Body length 6.0–6.4 mm, wing length 7.4–8.1 mm. Generally resembling male, coloration sometimes somewhat paler.

***Female terminalia*** (Fig. 3). Short and strongly modified. Tergite 9 ~ 2/3 of length and width of tergite 8 (Fig. 3A, C). Posterior margin of tergite 9 slightly concave. Tergite 10 and short, fleshy cerci fused, fused section sub-equal in length to tergite 9. Cerci appearing as two rounded lobes, with numerous setae at apex cerci widely separated with V-shape notch (Fig. 3A). Sternite 8 large, longer than tergites 8 and 9 together (Fig. 3C). Ventral margin of sternite 8 convex, posterolateral corner produced into finger-like process, covered with setae, and slanted upwards at ~ 45°, reaching further than cerci and hypogynial valves (Fig. 3C, D). Hypogynial valves short, subequal in length with tergite 10+cerci (Fig. 3A, C). Parts of genital chamber as genital fork, sternite 9 and genital opening fused and forming a complex strongly sclerotized structure (Fig. 3D). Area of genital opening pale, membranous, surrounded by more sclerotized sternite 9. Sternite 9 with numerous short setae. Posterior part of sternite 9 with a roughly triangular lobe between hypogynial valve and finger-like lobe of sternite 8, subequal in length to hypogynial valve. Anterior part of sternite 9 with a pair of invaginations lateral to genital fork, most probably holding the male gonostylus during the copulation. Genital fork narrowing to a point anteriorly, posteriorly fused with sternite 9 (Fig. 3D). Sternite 10 small, rounded.

***Egg.*** Dark, large, sub-equal in length of tergite 9, tergite 10 and cerci combined; shape oval, cross section roughly triangular.

##### Etymology.

The name of this small and unique species refers to the Neretva River (Fig. 4), one of the last pristine European rivers, from where it was collected in high numbers.

##### Distribution.

The new species is known from Bosnia and Herzegovina, Montenegro, and Slovenia (Fig. 5).

##### Remarks.

*Baeouraneretvaensis* sp. nov. is unique among the Western Palaearctic species. The closest related species is *Baeouramalickyi* Mendl & Tjeder, 1976, but *B.neretvaensis* can be differentiated from it by the long and slender gonostylus which terminates in a long seta (gonostylus more robust and flattened in *B.malickyi*, with only a short spine-like seta at tip), gonocoxite with a short, apical ventral lobe (long finger-like lobe in *B.malickyi*), a flat lobe at the base of the gonostylus present (no such lobe in *B.malickyi*) (Mendl and Tjeder 1976: figs 1–4). The female of the new species also differs from all described females by the presence of a pair of long finger-like lobes on sternite 8, which is longer than the hypogynial valve (a much shorter lobe is also present in female of *B.malickyi*; however, it is 1/2 as long as the hypogynial valve (Mendl and Tjeder 1976: figs 5–9).

### ﻿Faunistic records

#### ﻿Limoniidae

##### ﻿1. Antocha (Antocha) vitripennis

Taxon classificationAnimaliaDipteraLimoniidae

(Meigen, 1830)

D0F5A37D-8A3C-5241-AFD0-6487C0C5AEC0

###### Material examined.

**Croatia** • 1 male; Ličko-Senjska county, Rastovača, Tufa barrier Kozjak-Milanovac, Plitvice Lakes; 44.89416°N, 15.60888°E; alt. 545 m; 28 June 2017; emergence trap, P1; CKLP • 1 male, same locality, 31 May 2016; emergence trap, P5; leg. M. Ivković; UZC. **Slovenia** • 1 male; Savinjska, Ljubno ob Savinji; 46.332°N, 14.839°E; alt. 490 m; 23 July 2022; leg. M.C. de Haas; PCMCO.

###### Comments.

A common species. Larvae aquatic, associated with clear running water with rocky bottom.

##### ﻿2. Antocha (Orimargula) alpigena

Taxon classificationAnimaliaDipteraLimoniidae

(Mik, 1883)

FDB11FF7-945C-53D6-BDD1-7E29F0CA4D74

Fig. 6

###### Material examined.

**Bosnia and Herzegovina** • 6 males, 3 females; Krupac, Krupac 100–600 m from Neretva River upstream; 43.33092°N, 18.42894°E; alt. 805 m; 29 June 2022; leg. M. Ivković; 3 males and 2 females in CKLP, 3 males and 1 female in UZC. **Slovenia** • 1 male; Gozd Martuljek, Martuljški slapovi; 46.473611°N, 13.829333°E; alt. 850 m; 5 July 2022; leg. C. Quindroit; PCCQ.

###### Comments.

Much rarer species than *Antochavitripennis*, adults usually found along small, fast-flowing mountain rivers, streams, and waterfalls (Starý 2009). First record from Bosnia and Herzegovina.

**Figure 6.**
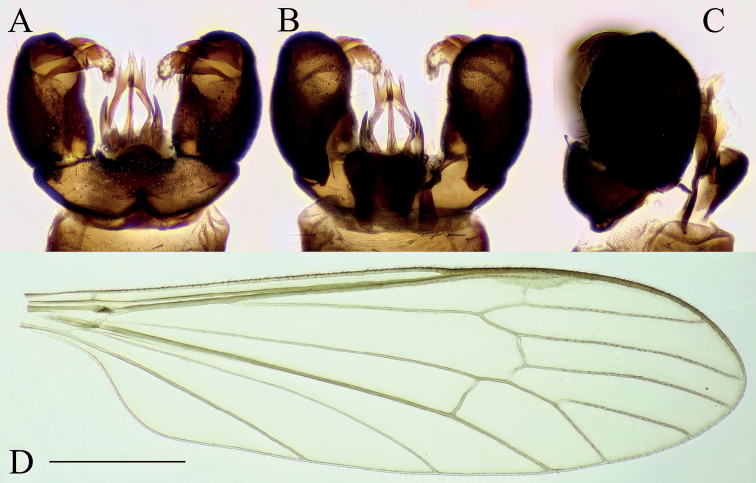
Antocha (Orimargula) alpigena, specimen: Bosnia and Herzegovina, Krupac, Krupac 100–600 m from Neretva River upstream (CKLP) **A–C** male terminalia **A** dorsal view **B** ventral view **C** lateral view **D** wing. Scale bar: 1 mm (**D**).

##### ﻿3. Atypophthalmus (Microlimonia) machidai

Taxon classificationAnimaliaDipteraLimoniidae

(Alexander, 1921)

93971630-DB0A-5332-A200-9121F60549A2

###### Material examined.

**Slovenia** • 1 male; Krma Valley; 46.370556°N, 13.88916°E; alt. 900 m; 6 July 2022; leg. C. Quindroit; PCCQ.

###### Comments.

A widely distributed species, known from the Palearctic and Oriental regions. It is relatively rare in Europe, and recently reported from Slovenia (Kolcsár et al. 2021).

##### ﻿4. Austrolimnophila (Austrolimnophila) ochracea

Taxon classificationAnimaliaDipteraLimoniidae

(Meigen, 1804)

85DE1CB8-07FF-58F4-93B1-87B2EF3453E0

###### Material examined.

**Slovenia** • 1 male; Ljubljana, castle; 46.048°N, 14.50977°E; alt. 340 m; 30 June 2022; leg. C. Quindroit; PCCQ.

###### Comments.

A common and widespread species in the Western Palaearctic.

##### ﻿5. Dicranomyia (Dicranomyia) chorea

Taxon classificationAnimaliaDipteraLimoniidae

(Meigen, 1818)

28C47AA8-F215-5758-BC2E-A4DA9853A3FD

Fig. 7

###### Material examined.

**Croatia** • 1 male; Ličko-Senjska county, Končarev Kraj, Spring of Bijela rijeka, Plitvice Lakes; 44.83472°N, 15.56194°E; alt. 720 m; 31 August 2015; emergence trap, P5 • 1 male, same locality, 31 October 2016; emergence trap, P6 • 1 male, same locality, 31 August 2021; emergence trap, P5; leg. M. Ivković; UZC • 1 male; Ličko-Senjska county, Rastovača, Tufa barrier Kozjak-Milanovac, Plitvice Lakes; 44.89416°N, 15.60888°E; alt. 545 m; 27 July 2017; emergence trap, P3; leg. M. Ivković; CKLP • 1 male, same locality; 28 June 2017; emergence trap, P1 • 2 males, 2 females; same locality; 26 July 2020; emergence trap, P1 • 7 males, 2 females; same locality; 28 June 2020; emergence trap, P1 • 2 males, 1 female; same locality; 31 August 2021; emergence trap, P1; leg. M. Ivković; CKLP.

**Figure 7.**
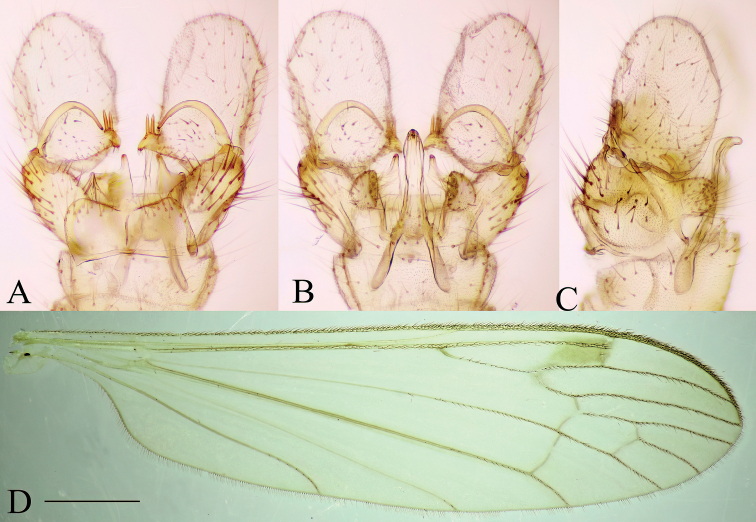
Dicranomyia (Dicranomyia) chorea, specimen: Croatia, spring of Bijela rijeka, Plitvice Lakes (CKLP) **A–C** male terminalia **A** dorsal view **B** ventral view **C** lateral view **D** wing. Scale bar: 1 mm (**D**).

###### Comments.

A very common and widespread species. Coloration of the specimens very variable from almost fully yellow to dark brown. All specimens examined are yellow.

##### ﻿6. Dicranomyia (Dicranomyia) conchifera

Taxon classificationAnimaliaDipteraLimoniidae

(Strobl, 1900)

92DA153C-87FF-5BA3-A71E-A58D401B9536

Fig. 8

###### Material examined.

**Croatia** • 1 male, 1 female; Ličko-Senjska county, Rastovača, Tufa barrier Kozjak-Milanovac, Plitvice Lakes; 44.89416°N, 15.60888°E; alt. 545 m; 30 June 2019; emergence trap, P3 • 4 males, 1 female; same locality; 28 May 2020; emergence trap, P3 • 1 female; same locality; 28 June 2020; emergence trap, P3 • 1 male, 1 female; same locality; 30 June 2021; emergence trap, P1 • 2 males, 4 females; same locality; 30 June 2021; emergence trap, P3; leg. M. Ivković; 2 males in UZC, other specimens in CKLP.

**Figure 8.**
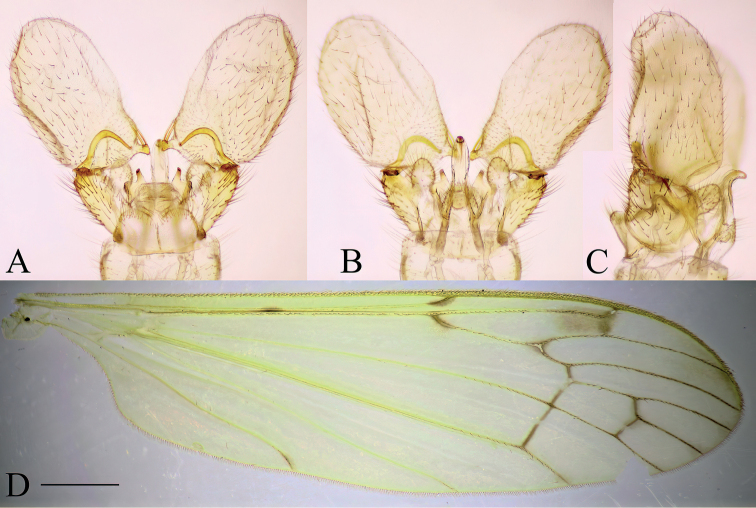
Dicranomyia (Dicranomyia) conchifera, specimen: Croatia, Tufa barrier Kozjak-Milanovac, Plitvice Lakes (CKLP) **A–C** male terminalia **A** dorsal view **B** ventral view **C** lateral view **D** wing. Scale bar: 1 mm (**D**).

###### Comments.

A rare and poorly known species. In the Balkans it is recorded from Albania, Bulgaria, Greece, North Macedonia, Serbia, and Slovenia. First records from Croatia.

##### ﻿7. Dicranomyia (Dicranomyia) didyma

Taxon classificationAnimaliaDipteraLimoniidae

(Meigen, 1804)

E0516930-94E3-55EC-9BAF-94C1043554A8

###### Material examined.

**Bosnia and Herzegovina** • 1 male, Izvor Ribnik; 44.40222°N, 16.80055°E; alt. 308 m; 24 April 2006; leg. M. Ivković; CKLP. **Croatia** • 1 male, 1 female; Ličko-Senjska county, Končarev Kraj, Spring of Bijela rijeka, Plitvice Lakes; 44.83472°N, 15.56194°E; alt. 720 m; 30 June 2015; emergence trap, P5 • 1 female; same locality; 27 July 2015; emergence trap, P5 • 13 females; same locality; 31 August 2015; emergence trap, P5 • 1 male, 7 females; same locality; 30 September 2015; emergence trap, P5 • 12 males, 6 females; same locality; 31 August 2016; emergence trap, P5 • 5 males, 3 females; same locality; 29 September 2016; emergence trap, P5 • 8 females; same locality; 28 June 2017; emergence trap, P5 • 7 males; same locality; 28 June 2017; emergence trap, P5 • 1 male; same locality; 28 June 2017; emergence trap, P6 • 3 males, 6 females; same locality; 27 July 2017; emergence trap, P5 • 4 males, 1 female; same locality; 31 August 2017; emergence trap, P5 • 1 male; same locality; 29 September 2017; emergence trap, P5 • 1 male, 1 female; same locality; 26 July 2018; emergence trap, P5 • 1 male, 1 female; same locality; 31 August 2018; emergence trap, P5 • 1 female; same locality; 31 August 2018; emergence trap, P6 • 6 males, 1 female; same locality; 31 August 2018; emergence trap, P5 • 2 males, 1 female; same locality; 30 September 2018; emergence trap, P5 • 1 male, 1 female; same locality; 30 June 2019; emergence trap, P5 • 2 males; same locality; 30 June 2019; emergence trap, P5 • 3 males, 5 females; same locality; 27 July 2019; emergence trap, P5 • 1 female; same locality; 30 August 2019; emergence trap, P4 • 2 males, 3 females; same locality; 30 August 2019; emergence trap, P5 • 6 males, 1 female; same locality; 30 September 2019; emergence trap, P5 • 1 male, 1 female; same locality; 29 October 2019; emergence trap, P5 • 2 females; same locality; 26 July 2020; emergence trap, P5 • 1 male; same locality; 29 September 2021; emergence trap, P5; leg. M. Ivković; CKLP • 1 female; Ličko-Senjska county, Plitvica Selo, Stream Plitvica, Plitvice Lakes; 44.90222°N, 15.6075°E; alt. 555 m; 25 July 2008; emergence trap, P6 • 1 female; same locality; 25 July 2008; emergence trap, P3; leg. M. Ivković; CKLP • 1 female; Ličko-Senjska county, Plitvički Ljeskovac, Tufa barrier Labudovac, Plitvice Lakes; 44.87138°N, 15.59972°E; alt. 630 m; 30 October 2014; emergence trap, P3; leg. M. Ivković; CKLP • 2 females; Ličko-Senjska county, Rastovača, Tufa barrier Kozjak-Milanovac, Plitvice Lakes; 44.89416°N, 15.60888°E; alt. 545 m; 30 June 2015; emergence trap, P3 • 5 males, 1 female; same locality; 31 May 2016; emergence trap, P3; leg. M. Ivković; CKLP • 5 males, 1 female; same locality; 31 May 2016; emergence trap, P3 • 1 male; same locality; 30 June 2016; emergence trap, P3 • 2 males, 2 females; same locality; 25 July 2016; emergence trap, P2 • 1 male; same locality; 25 July 2016; emergence trap, P5 • 1 male; same locality; 29 May 2018; emergence trap, P3 • 3 males, 6 females; same locality; 30 August 2019; emergence trap, P4; leg. M. Ivković; 2 males and 2 females in UZC, other specimens in CKLP.

###### Comments.

A widely distributed species, associated with small rivers and streams. The most abundant species in the emergence trap samples from Croatia.

##### ﻿8. Dicranomyia (Dicranomyia) imbecilla

Taxon classificationAnimaliaDipteraLimoniidae

Lackschewitz, 1941

F7AE6208-7A6B-5E65-A771-057DA57AE4A9

###### Material examined.

**Albania** • 1 male; Dibër, Fushë-Bulqizë; 41.5279°N, 20.2983°E; alt. 780 m; light trap; 26 July 2021; leg. A. de Ketelaere; PCMCO. **Slovenia** • 1 male; Gorenjska, Gozd Martuljek, Juliske alpe, River Sava; 46.483°N, 13.838°E; alt. 745 m; light trap; leg. M.C. d’Oliveira; PCMCO • 1 male; Stara fužina, Mostnica river; 46.297889°N, 13.886389°E; alt. 600 m; 3 July 2022; leg. C. Quindroit; PCCQ.

###### Comments.

A poorly known species, only recently reinstated as a valid species and probably a more widely distributed species than thought (Starý and Stubbs 2015). Here we present the first records from Albania and Slovenia.

##### ﻿9. Dicranomyia (Dicranomyia) mitis

Taxon classificationAnimaliaDipteraLimoniidae

(Meigen, 1830)

C52E7D81-988D-57B0-BABF-99B23BB05FFE

###### Material examined.

**Croatia** • 1 male, 1 female; Ličko-Senjska county, Končarev Kraj, Spring of Bijela rijeka, Plitvice Lakes; 44.83472°N, 15.56194°E; alt. 720 m; 30 June 2015; emergence trap, P5; leg. M. Ivković; CKLP.

###### Comments.

Common species with a wide distribution across Europe.

##### ﻿10. Dicranomyia (Dicranomyia) quadra

Taxon classificationAnimaliaDipteraLimoniidae

(Meigen, 1838)

D52954D8-0D3C-518C-8677-876940C34F63

Fig. 9

###### Material examined.

**Croatia** • 1 male; Ličko-Senjska county, Končarev Kraj, Spring of Bijela rijeka, Plitvice Lakes; 44.83472°N, 15.56194°E; alt. 720 m; 28 June 2017; emergence trap, P3 • 2 males; same locality; 28 June 2017; emergence trap, P5 • 1 female; same locality; 28 June 2017; emergence trap, P6 • 1 male; same locality; 29 May 2018; emergence trap, P5 • 1 male; same locality; 29 May 2018; emergence trap, P4 • 1 female; same locality; 29 June 2018; emergence trap, P4 • 1 female; same locality; 30 June 2019; emergence trap, P5 • 2 males; same locality; 30 June 2019; emergence trap, P6 • 2 males, 1 female; same locality; 27 July 2019; emergence trap, P6 • 1 female; same locality; 27 July 2019; emergence trap, P4 • 2 males, 2 females; same locality; 28 May 2020; emergence trap, P6 • 1 male; same locality; 30 June 2021; emergence trap, P5 • 1 male; same locality; 30 June 2021; emergence trap, P6; leg. M. Ivković; CKLP • 1 male; Ličko-Senjska county, Plitvički Ljeskovac, Tufa barrier Labudovac, Plitvice Lakes; 44.87138°N, 15.59972°E; alt. 630 m; 29 May 2021; emergence trap, P6; leg. M. Ivković; 2 males and 2 females in UZC, other specimens in CKLP.

**Figure 9.**
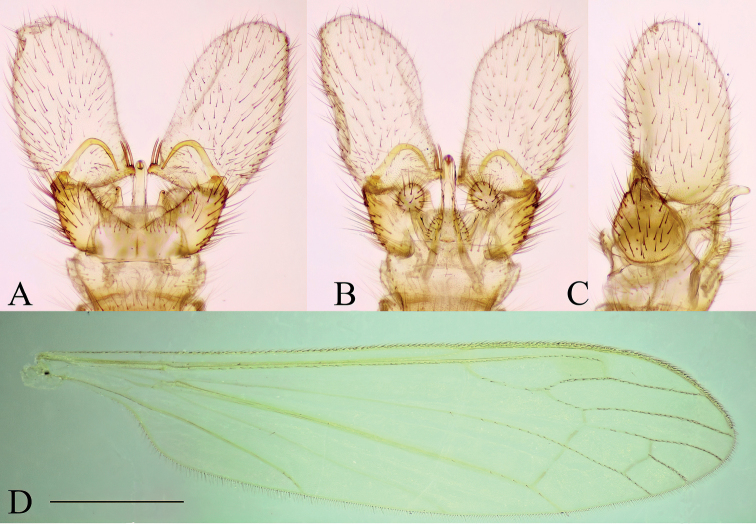
Dicranomyia (Dicranomyia) quadra, specimen: Croatia, spring of Bijela rijeka, Plitvice Lakes (CKLP) **A–C** male terminalia **A** dorsal view **B** ventral view **C** lateral view **D** wing. Scale bar: 1 mm (**D**).

###### Comments.

A poorly known species and only recently reinstated as a valid species (Starý and Stubbs 2015). It is known from several countries from the Balkans, and we present the first records from Croatia.

##### ﻿11. Dicranomyia (Melanolimonia) morio

Taxon classificationAnimaliaDipteraLimoniidae

(Fabricius, 1787)

9C7F703B-A83A-5B3A-8370-295EFF9EE188

Fig. 10

###### Material examined.

**Croatia** • 1 female; Ličko-Senjska county, Končarev Kraj, Spring of Bijela rijeka, Plitvice Lakes; 44.83472°N, 15.56194°E; alt. 720 m; 30 August 2020; emergence trap, P4 • 5 males, 1 female; same locality; 28 September 2020; emergence trap, P5; leg. M. Ivković; 2 males in UZC, other specimens in CKLP.

###### Comments.

Widely distributed species in the Palearctic, adults usually found around springs and smaller streams, larvae probably live on moss covered wet rocks. First records from Croatia.

**Figure 10.**
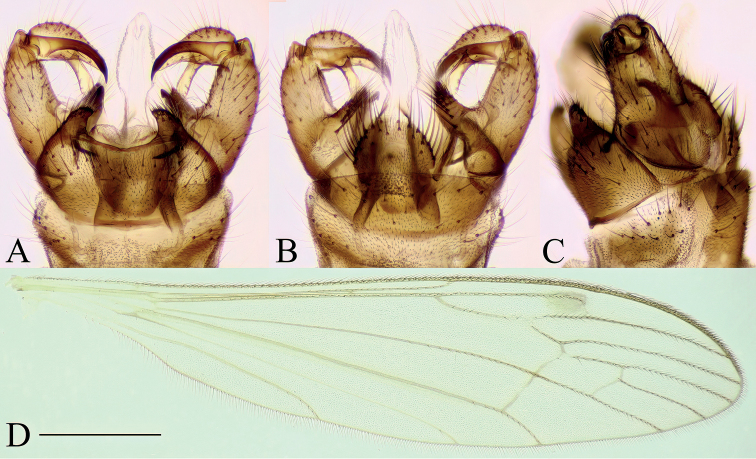
Dicranomyia (Melanolimonia) morio, specimen: Croatia, spring of Bijela rijeka, Plitvice Lakes (CKLP) **A–C** male terminalia **A** dorsal view **B** ventral view **C** lateral view **D** wing. Scale bar: 1 mm (**D**).

##### ﻿12. Dicranomyia (Melanolimonia) stylifera

Taxon classificationAnimaliaDipteraLimoniidae

Lackschewitz, 1928

EA7B6210-D800-515F-BF5D-16EED7BB6C2F

###### Material examined.

**Slovenia** • 1 male; Mojstrana, Slap Peričnik; 46.439111°N, 13.894278°E; alt. 900 m; 05 july 2022; leg C. Quindroit; PCCQ.

###### Comments.

A rare and poorly known species, associated with calcareous habitats (Salmela 2008; Stubbs 2021).

##### ﻿13. Dicranomyia (Numantia) fusca

Taxon classificationAnimaliaDipteraLimoniidae

(Meigen, 1804)

D3077040-58B5-548F-8424-5149C9701557

###### Material examined.

**Slovenia** • 2 males; Savinjska, Ljubno ob Savinji; 46.332°N, 14.839°E; alt. 490 m; 23 July 2022; leg. M.C. de Haas; PCMCO.

###### Comments.

Common species and widely distributed in the Holarctic.

##### ﻿14. Dicranomyia (Sivalimnobia) aquosa

Taxon classificationAnimaliaDipteraLimoniidae

Verrall, 1886

F5706036-2EDE-5034-B3C6-509399C87318

###### Material examined.

**Slovenia** • 3 males, 1 female; Gozd Martuljek, Martuljški slapovi; 46.473611°N, 13.829333°E; alt. 850 m; 5 July 2022; leg. C. Quindroit; PCCQ • 1 male; Mojstrana, Slap Peričnik; 46.439111°N, 13.894278°E; alt. 900 m; 5 July 2022; leg C. Quindroit; PCCQ.

###### Comments.

A rare species associated with hygropetric habitats, usually found around waterfalls. Larvae most probably live on the surface of permanently wet rocks (Stubbs 2021).

##### ﻿15. Dicranophragma (Brachylimnophila) nemorale

Taxon classificationAnimaliaDipteraLimoniidae

(Meigen, 1818)

AF52A666-B813-54AB-9BEF-ED82BADC55A7

###### Material examined.

**Croatia** • 2 males; Ličko-Senjska county, Plitvički Ljeskovac, Tufa barrier Labudovac, Plitvice Lakes; 44.87138°N, 15.59972°E; alt. 630 m; 29 September 2021; emergence trap, P6; leg. M. Ivković; CKLP • 1 male; Ličko-Senjska county, Rastovača, Tufa barrier Kozjak-Milanovac, Plitvice Lakes; 44.89416°N, 15.60888°E; alt. 545 m; 29 September 2017; emergence trap, P4; leg. M. Ivković; 1 male in UZC, other specimens in CKLP. **Slovenia** • 1 male; Stara fužina, Mostnica river; 46.297889°N, 13.886389°E; alt. 600 m; 3 July 2022; leg. C. Quindroit; PCCQ.

###### Comments.

Common.

##### ﻿16. Dicranoptycha fuscescens

Taxon classificationAnimaliaDipteraLimoniidae

(Schummel, 1829)

3F939DCA-A562-5600-9B26-025192CF41E9

###### Material examined.

**Slovenia** • 1 male; Bovec, junction Soča and Koritnica rivers; 46.330167°N, 13.577028°E; alt. 400 m; 7 July 2022; leg. C. Quindroit; PCCQ.

###### Comments.

A common species with wide distribution range in the Palaearctic.

##### ﻿17. Elliptera omissa

Taxon classificationAnimaliaDipteraLimoniidae

Schiner, 1863

94D0ADA4-E08A-501A-B076-AE9A84C12B4D

###### Material examined.

**Croatia** • 1 male; Primorsko-Goranska county, Gorski Kotar, Spring of River Kupa and just below the spring; 45.4919°N, 14.6925°E; alt. 756 m; 06 August 2021; leg. M. Ivković; CKLP. **Slovenia** • 1 male; Trenta, Soča source; 46.411972°N, 13.729583°E; alt. 950 m; 7 July 2022; leg. C. Quindroit; PCCQ.

###### Comments.

A rare species, known only from a few European countries, recently reported from Croatia (Kolcsár et al. 2015a). The species is connected to hygropetric habitats, usually found around waterfalls and fast-flowing rocky mountain streams and rivers.

##### ﻿18. Ellipteroides (Ellipteroides) lateralis

Taxon classificationAnimaliaDipteraLimoniidae

(Macquart, 1835)

252CD541-D918-5AE4-93F6-47470CB7947C

###### Material examined.

**Croatia** • 1 male; Ličko-Senjska county, Rastovača, Tufa barrier Kozjak-Milanovac, Plitvice Lakes; 44.89416°N, 15.60888°E; alt. 545 m; 29 May 2018; emergence trap, P6; leg. M. Ivković; CKLP.

###### Comments.

Species usually found around streams and rivers with sandy sediments.

##### ﻿19. Ellipteroides (Protogonomyia) alboscutellatus

Taxon classificationAnimaliaDipteraLimoniidae

(von Roser, 1840)

39C058AF-DB98-51CB-8580-C90A5A338BE1

Fig. 11

###### Material examined.

**Bosnia and Herzegovina** • 1 male, 1 female; Krupac, Krupac 100–600 m from Neretva River upstream; 43.33092°N, 18.42894°E; alt. 805 m; 29 June 2022; leg. M. Ivković; CKLP. **Slovenia** • 1 male, 2 females; Vintgar gorge; 46.393333°N, 14.086056°E; alt. 600 m; 1 July 2022; leg C. Quindroit; PCCQ • 5 males; Stara fužina, Mostnica river; 46.297889°N, 13.886389°E; alt. 600 m; 3 July 2022; leg. C. Quindroit; PCCQ.

**Figure 11.**
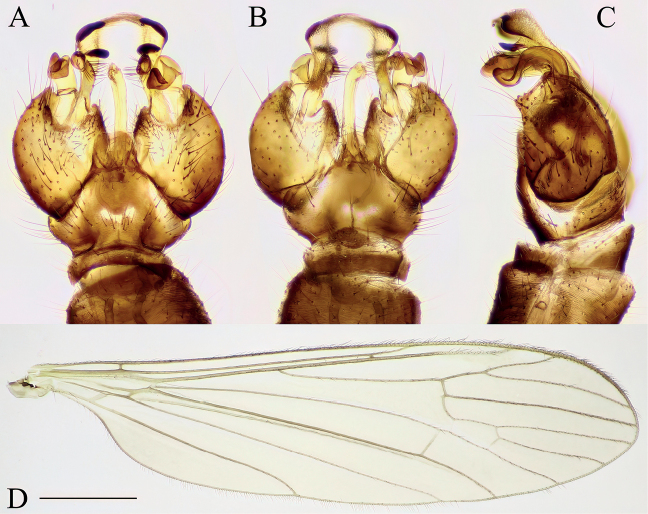
Ellipteroides (Protogonomyia) alboscutellatus, specimen: Bosnia and Herzegovina, Krupac, Krupac 100–600 m from Neretva River upstream (CKLP) **A–C** male terminalia **A** dorsal view **B** ventral view **C** lateral view **D** wing. Scale bar: 1 mm (**D**).

###### Comments.

First record from Bosnia and Herzegovina.

##### ﻿20. Ellipteroides (Protogonomyia) limbatus

Taxon classificationAnimaliaDipteraLimoniidae

(von Roser, 1840)

8D26D8A1-AF01-5DC1-B042-A45EF5A29298

Fig. 12

###### Material examined.

**Bosnia and Herzegovina** • 2 females; Krupac, Krupac 100–600 m from Neretva River upstream; 43.33092°N, 18.42894°E; alt. 805 m; 29 June 2022; leg. M. Ivković; CKLP. **Montenegro** • 2 males; Mouth of Stream Desna rijeka to River Mojanska rijeka; 42.68888°N, 19.72777°E; alt. 925 m; 08 July 2012; leg. M. Ivković; CKLP • 1 male; River Murinska Rijeka; 42.6525°N, 19.88361°E; alt. 1000 m; 07 July 2012; leg. M. Ivković; 1 male and 1 female in UZC, other specimens in CKLP. **Slovenia** • 1 male, 1 female; Savinjska, Ljubno ob Savinji; 46.332°N, 14.839°E; alt. 490 m; 23 July 2022; leg. M.C. de Haas; PCMCO.

**Figure 12.**
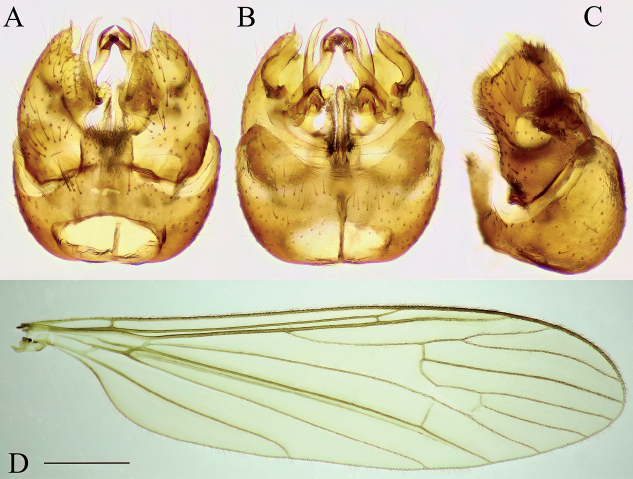
Ellipteroides (Protogonomyia) limbatus, specimen: Montenegro, mouth of stream Desna rijeka to River Mojanska Rijeka (CKLP) **A–C** male terminalia **A** dorsal view **B** ventral view **C** lateral view **D** wing. Scale bar: 1 mm (**D**).

###### Comments.

First record from Bosnia and Herzegovina.

##### ﻿21. Eloeophila miliaria

Taxon classificationAnimaliaDipteraLimoniidae

(Egger, 1863)

A5DA6322-B007-5815-821D-BEA6E7380F99

Fig. 13

###### Material examined.

**Bosnia and Herzegovina** • 1 male; Ulogski Buk, Ulogski Buk on Neretva; 43.40467°N, 18.32423°E; alt. 675 m; 01 July 2022; leg. M. Ivković; CKLP.

###### Comments.

A relatively rare species, and only recently reported from Croatia (Kolcsár et al. 2015a); here we report the species for the first time from Bosnia and Herzegovina.

**Figure 13.**
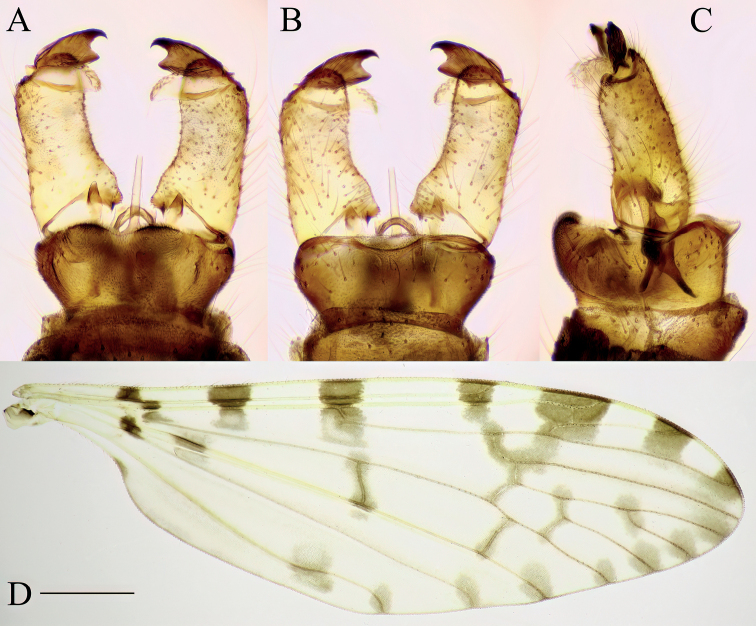
*Eloeophilamiliaria*, specimen: Bosnia and Herzegovina, Ulogski Buk, Ulogski Buk on Neretva (CKLP) **A–C** male terminalia **A** dorsal view **B** ventral view **C** lateral view **D** wing. Scale bar: 1 mm (**D**).

##### ﻿22. Erioptera (Erioptera) lutealutea

Taxon classificationAnimaliaDipteraLimoniidae

Meigen, 1804

9BA3CBAE-7D6A-5AB2-B1D6-9BEB0D2354F9

###### Material examined.

**Slovenia** • 1 male; Savinjska, Ljubno ob Savinji; 46.333°N, 14.8387°E; alt. 745 m; light trap; 20 August 2019; leg. M.C. d’Oliveira; PCMCO.

###### Comments.

A common species with wide distribution range in the Palaearctic.

##### ﻿23. Gonomyia (Prolipophleps) abbreviata

Taxon classificationAnimaliaDipteraLimoniidae

Loew, 1873

780AB438-C990-57BC-B8AB-ABDEF59EB748

###### Material examined.

**Slovenia** • 1 male; Savinjska, Ljubno ob Savinji; 46.332°N, 14.839°E; alt. 490 m; 22 July 2022; light trap; leg. M.C. de Haas; PCMCO.

###### Comments.

A relatively rare and poorly known species with a wide distribution range from Iran to Great Britain; however, only with scattered records. First record from Slovenia.

##### ﻿24. Helius (Helius) pallirostris

Taxon classificationAnimaliaDipteraLimoniidae

Edwards, 1921

BB424974-AB75-5AC0-91BE-2836A8B6CD59

###### Material examined.

**Croatia** • 1 female; Ličko-Senjska county, Plitvički Ljeskovac, Tufa barrier Labudovac, Plitvice Lakes; 44.87138°N, 15.59972°E; alt. 630 m; 27 July 2019; emergence trap, P1; leg. M. Ivković; CKLP.

###### Comments.

A relatively common species in Central Europe; however, this is the first record from Croatia and from the Western Balkans.

##### ﻿25. Hexatoma (Eriocera) chirothecata

Taxon classificationAnimaliaDipteraLimoniidae

(Scopoli, 1763)

0EE7FDC6-D570-5302-8006-975E3B001E9D

###### Material examined.

**Croatia** • 1 male; Ličko-Senjska county, Končarev Kraj, Spring of Bijela rijeka, Plitvice Lakes; 44.83472°N, 15.56194°E; alt. 720 m; 26 July 2020; emergence trap, P6; leg. M. Ivković; CKLP • 1 male; Ličko-Senjska county, Plitvički Ljeskovac, Tufa barrier Labudovac, Plitvice Lakes; 44.87138°N, 15.59972°E; alt. 630 m; 25 July 2014; emergence trap, P2 • 2 males; same locality; 27 July 2015; emergence trap, P2 • 1 female; same locality; 27 July 2015; emergence trap, P7 • 3 females; same locality; 30 June 2021; emergence trap, P6 • 1 male, 1 female; same locality; 30 June 2021; emergence trap, P6 • 2 males; same locality; 31 August 2021; emergence trap, P3; leg. M. Ivković; 1 male and 1 female in UZC, other specimens in CKLP • 1 male; Ličko-Senjska county, Rastovača, Tufa barrier Kozjak-Milanovac, Plitvice Lakes; 44.89416°N, 15.60888°E; alt. 545 m; 30 August 2019; emergence trap, P5; leg. M. Ivković; CKLP. **Slovenia** • 1 female; Savinjska, Ljubno ob Savinji; 46.332°N, 14.839°E; alt. 490 m; 16 July 2022; light trap; leg. M.C. de Haas; PCMCO.

###### Comments.

A poorly known species. It seems common in the Western Balkans, reported from all countries; however, it is relatively rare in other parts of Europe. The species seems to prefer small calcareous rivers and streams.

##### ﻿26. Hexatoma (Hexatoma) bicolor

Taxon classificationAnimaliaDipteraLimoniidae

(Meigen, 1818)

77125844-5A54-5C2D-A3A2-1820EC137BE1

###### Material examined.

**Slovenia** • 1 male; Savinjska, Luče; 46.357°N, 14.753°E; alt. 515 m; 20 July 2022; leg. M.C. de Haas; PCMCO.

###### Comments.

A poorly known species associated with streams and rivers with sandy or gravelly banks (Starý 2009).

##### ﻿27. Idiocera (Euptilostena) jucunda

Taxon classificationAnimaliaDipteraLimoniidae

(Loew, 1873)

F2E7F58E-A78F-56DA-8349-A5809ADA3946

Fig. 14

###### Material examined.

**Bosnia and Herzegovina** • 1 male, 4 females; Sutjeska, Jabučica Stream; 43.29°N, 18.6172°E; alt. 767 m; 04 July 2012; UV Lamp; leg. M. Ivković; CKLP • 1 male; Ulog, Neretva at Ulog Camp site; 43.41714°N, 18.31205°E; alt. 650 m; 28 June 2022; leg. W. Graf; CKLP. **Slovenia** • 1 male; Gorenjska, Gozd Martuljek, Juliske alpe, River Sava; 46.483°N, 13.838°E; alt. 745 m; 20 August 2019; light trap; leg. M.C. d’Oliveira; PCMCO.

**Figure 14.**
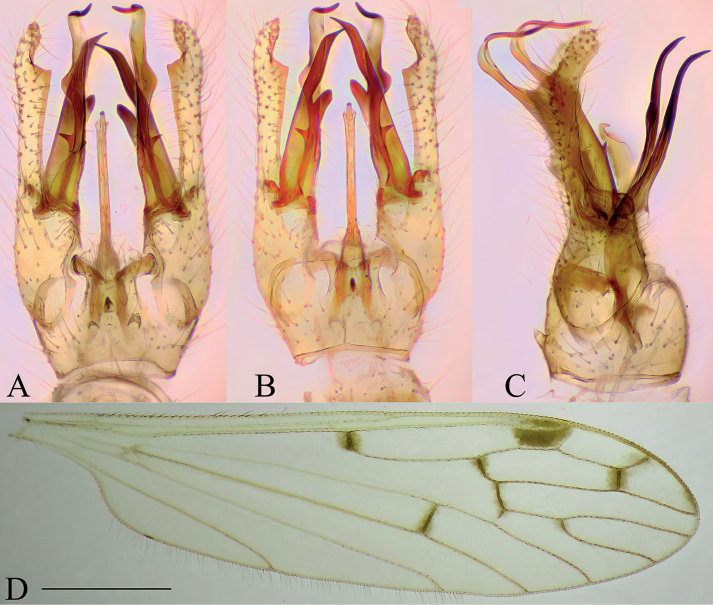
Idiocera (Euptilostena) jucunda, specimen: Bosnia and Herzegovina, Ulog, Neretva at Ulog Camp site (CKLP) **A–C** male terminalia **A** dorsal view **B** ventral view **C** lateral view **D** wing. Scale bar: 1 mm (**D**).

###### Comments.

A rare and poorly known species associated with mountain rivers and streams with sandy and gravelly banks (Starý 2009). We collected the species near similar habitats, with light traps. First records from Bosnia and Herzegovina.

##### ﻿28. Idiocera (Idiocera) lackschewitzi

Taxon classificationAnimaliaDipteraLimoniidae

(Starý, 1977)

6EA80BA9-9C3F-53AF-B163-E64A4BB176E2

Fig. 15

###### Material examined.

**Bosnia and Herzegovina** • 1 female; Sutjeska, Jabučica Stream; 43.29°N, 18.6172°E; alt. 767 m; 04 July 2012; leg. M. Ivković; CKLP • 1 male; Ulog, Neretva at Ulog Camp site; 43.41714°N, 18.31205°E; alt. 650 m; 28 June 2022; leg. W. Graf; CKLP.

###### Comments.

A very rare species, only known from Albania, Greece, Italy (Sicily), and North Macedonia. First records from Bosnia and Herzegovina. Specimens collected along or near rivers with gravelly banks, usually together with *Idiocerajucunda*.

**Figure 15.**
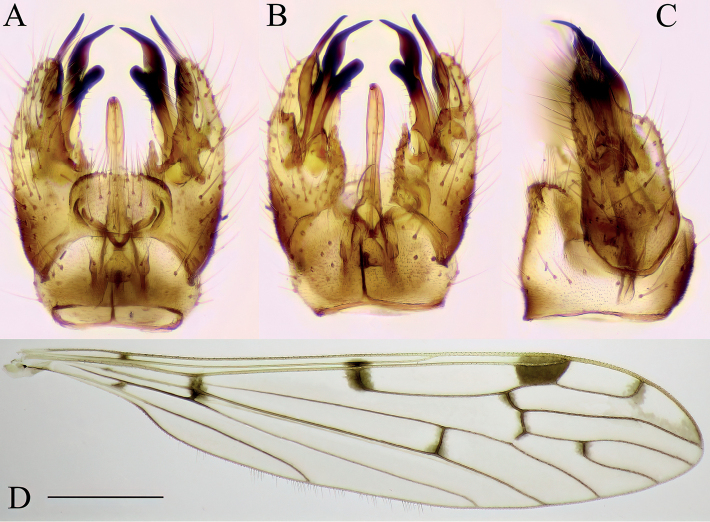
Idiocera (Idiocera) lackschewitzi, specimen: Bosnia and Herzegovina, Ulog, Neretva at Ulog Camp site (CKLP) **A–C** male terminalia **A** dorsal view **B** ventral view **C** lateral view **D** wing. Scale bar: 1 mm (**D**).

##### ﻿29. Ilisia maculata

Taxon classificationAnimaliaDipteraLimoniidae

(Meigen, 1804)

125C731C-3566-54C6-9524-4E69D9C42FC3

###### Material examined.

**Slovenia** • 1 male; Savinjska, Ljubno ob Savinji; 46.332°N, 14.839°E; alt. 490 m; 22 July 2022; light trap; leg. M.C. de Haas; PCMCO.

###### Comments.

A common species, associated with rich organic muds (Podeniene 2009), and can be found around various water bodies. First record from Slovenia.

##### ﻿30. Limonia nubeculosa

Taxon classificationAnimaliaDipteraLimoniidae

Meigen, 1804

1EBF1B58-BD35-5ED6-B8B5-93896A2EFB10

###### Material examined.

**Croatia** • 1 female; Ličko-Senjska county, Končarev Kraj, Spring of Bijela rijeka, Plitvice Lakes; 44.83472°N, 15.56194°E; alt. 720 m; 29 May 2018; emergence trap, P5; leg. M. Ivković; CKLP • 1 female; Zagreb, Stream Jelenovac; 45.82306°N, 15.95235°E; alt. 170 m; 10 April 2020; leg. M. Ivković (not stored)

###### Comments.

A very common and widely distributed species.

##### ﻿31. Limonia sylvicola

Taxon classificationAnimaliaDipteraLimoniidae

(Schummel, 1829)

B4B1CB7D-CCC0-5304-9F28-D0C4475ECE92

###### Material examined.

**Slovenia** • 1 male; Stara fužina, Mostnica river; 46.297889°N, 13.886389°E; alt. 600 m; 3 July 2022; leg. C. Quindroit; PCCQ.

###### Comments.

A common species.

##### ﻿32. Limonia taurica

Taxon classificationAnimaliaDipteraLimoniidae

(Strobl, 1895)

C0B7E083-3511-5791-BC97-6E9EDB708B92

###### Material examined.

**Slovenia** • 1 male; Stara fužina, Puncrat; 46.303778°N, 13.839917°E; alt. 1400 m; 2 July 2022; leg. C. Quindroit; PCCQ.

###### Comments.

A widely distributed species, known from several European countries and from China.

##### ﻿33. Limonia phragmitidis

Taxon classificationAnimaliaDipteraLimoniidae

(Schrank, 1781)

32328B44-FEEF-5CA4-8B80-40A0769ED08C

###### Material examined.

**Croatia** • 1 male; Zagreb, Stream Jelenovac; 45.82306°N, 15.95235°E; alt. 170 m; 10 April 2020; leg. M. Ivković; CKLP.

###### Comments.

A very common and widely distributed species.

##### ﻿34. Lipsothrix nobilis

Taxon classificationAnimaliaDipteraLimoniidae

Loew, 1873

BCF4F624-60FC-5B5C-B850-E23F8488F843

###### Material examined.

**Croatia** • 1 male; Ličko-Senjska county, Plitvički Ljeskovac, Tufa barrier Labudovac, Plitvice Lakes; 44.87138°N, 15.59972°E; alt. 630 m; 29 May 2015; emergence trap, P5; leg. M. Ivković; CKLP.

###### Comments.

A common Western Palaearctic species, larvae and pupae associated with partly submerged decaying larger wood in streams and rivers (Hancock et al. 2009).

##### ﻿35. Lipsothrix remota

Taxon classificationAnimaliaDipteraLimoniidae

(Walker, 1848)

378A836B-0CB9-59B9-AD81-1E45C62BCA17

###### Material examined.

**Croatia** • 1 male; Ličko-Senjska county, Končarev Kraj, Spring of Bijela rijeka, Plitvice Lakes; 44.83472°N, 15.56194°E; alt. 720 m; 30 June 2021; emergence trap, P5; leg. M. Ivković; UZC • 1 male; Ličko-Senjska county, Plitvički Ljeskovac, Spring of Crna rijeka, Plitvice Lakes; 44.83714°N, 15.60752°E; alt. 680 m; 30 June 2021; emergence trap, P6 • 1 female; same locality; 29 September 2021; emergence trap, P5; leg. M. Ivković; CKLP • 1 male; Ličko-Senjska county, Plitvički Ljeskovac, Tufa barrier Labudovac, Plitvice Lakes; 44.87138°N, 15.59972°E; alt. 630 m; 30 June 2014; emergence trap, P4 • 1 male; same locality; 30 June 2015; emergence trap, P6; leg. M. Ivković; CKLP.

###### Comments.

A common European species with similar habitat reference as *Lipsothrixnobilis* (Hancock et al. 2009).

##### ﻿36. Molophilus (Molophilus) aduncus

Taxon classificationAnimaliaDipteraLimoniidae

Starý, 1978

3F2D3D44-5D69-53AE-997D-6A0D672861CC

###### Material examined.

**Albania** • 1 male; Dibër, Fushë-Bulqizë; 41.5279°N, 20.2983°E; alt. 780 m; 26 July 2021; light trap; leg. A. de Ketelaere; PCMCO.

###### Comments.

A very rare species, reported only from Andorra, Bulgaria, Spain, and Russia: North Caucasus in Europe. First record from Albania.

##### ﻿37. Molophilus (Molophilus) appendiculatus

Taxon classificationAnimaliaDipteraLimoniidae

(Staeger, 1840)

903B5242-0593-5E23-B69C-E7E5CDFA8433

###### Material examined.

**Croatia** • 1 male; Krapinsko-Zagorska county, Mountain Ivanščica, Spring Podbel; 46.20111°N, 16.25611°E; alt. 346 m; 15 July 2014; leg. M. Ivković; CKLP. **Slovenia** • 2 males; Vintgar gorge; 46.39333°N, 14.086056°E; alt. 600 m; 1 July 2022; leg C. Quindroit; PCCQ.

###### Comments.

Common species that occurs in several terrestrial and semi-aquatic habitats.

##### ﻿38. Molophilus (Molophilus) bifidus

Taxon classificationAnimaliaDipteraLimoniidae

Goetghebuer, 1920

783ED399-FDF1-5D3A-A812-337E55F6C1E4

###### Material examined.

**Albania** • 3 males; Dibër, Fushë-Bulqizë; 41.5279°N, 20.2983°E; alt. 780 m; 26 July 2021; light trap; leg. A. de Ketelaere; PCMCO.

###### Comments.

A common species, associated with springs and small headwaters.

##### ﻿39. Molophilus (Molophilus) corniger

Taxon classificationAnimaliaDipteraLimoniidae

de Meijere, 1920

4757D6E6-41BA-5BD7-9B83-E4716D0FC9D8

###### Material examined.

**Croatia** • 1 male; Ličko-Senjska county, Plitvički Ljeskovac, Tufa barrier Labudovac, Plitvice Lakes; 44.87138°N, 15.59972°E; alt. 630 m; 30 June 2014; emergence trap, P4; leg. M. Ivković; CKLP. **Slovenia** • 1 male; Savinjska, Ljubno ob Savinji; 46.332°N, 14.839°E; alt. 490 m; 23 July 2022; leg. M.C. de Haas; PCMCO.

###### Comments.

Common European species; however, these are the first records from Croatia and Slovenia.

##### ﻿40. Molophilus (Molophilus) crassipygus

Taxon classificationAnimaliaDipteraLimoniidae

de Meijere, 1918

47C41BFB-CE35-55D4-B85C-DA5F4ECE5CF6

###### Material examined.

**Bosnia and Herzegovina** • 1 male; Ulog, Neretva at Ulog Camp site; 43.41714°N, 18.31205°E; alt. 650 m; 28 June 2022; leg. W. Graf; CKLP.

###### Comments.

Another relatively common European species, which is recently reported from several countries from the Balkan (Kolcsár et al. 2015b, 2021). Here we report it for the first time from Bosnia and Herzegovina.

##### ﻿41. Molophilus (Molophilus) lackschewitzianus

Taxon classificationAnimaliaDipteraLimoniidae

Alexander, 1953

3D016FBC-9D33-58D1-B099-D41BEECF0110

###### Material examined.

**Slovenia** • 3 males; Vintgar gorge; 46.39333°N, 14.086056°E; alt. 600 m; 1 July 2022; leg C. Quindroit; PCCQ.

###### Comments.

A relatively rare species, associated with calcareous habitats (Stubbs 2021), but rarely it can also be found around non-calcareous streams (Kolcsár and Soltész 2018).

##### ﻿42. Molophilus (Molophilus) medius

Taxon classificationAnimaliaDipteraLimoniidae

de Meijere, 1918

2F52E04B-427F-5100-A070-77DD7FA84475

###### Material examined.

**Slovenia** • 3 males, 1 female; Savinjska, Ljubno ob Savinji; 46.332°N, 14.839°E; alt. 490 m; 8 August 2020; light trap; leg. M.C. de Haas; PCMCO • 1 male; Savinjska, Ljubno ob Savinji; 46.332°N, 14.839°E; alt. 490 m; 22 July 2022; light trap; leg. M.C. de Haas; PCMCO.

###### Comments.

A common and widespread species in Europe, here reported for the first time from Slovenia.

##### ﻿43. Molophilus (Molophilus) propinquus

Taxon classificationAnimaliaDipteraLimoniidae

(Egger, 1863)

B1677623-C8F6-5F58-9431-D94C82E9E291

###### Material examined.

**Bosnia and Herzegovina** • 1 male; Ulog, Ulog on Neretva River; 43.42414°N, 18.30837°E; alt. 640 m; 29 June 2022; leg. W. Graf; CKLP.

###### Comments.

Common and usually abundant species around wet habitats, especially along rivers and streams.

##### ﻿44. Molophilus (Molophilus) pullus

Taxon classificationAnimaliaDipteraLimoniidae

Lackschewitz, 1927

6769F753-7CF4-5FA8-8D7E-9425792826D1

Fig. 16

###### Material examined.

**Croatia** • 1 male, 2 females; Zagreb, Stream Jelenovac; 45.82306°N, 15.95235°E; alt. 170 m; 10 April 2020; leg. M. Ivković; CKLP.

###### Comments.

A rarer *Molophilus* species, known only from few European countries. First record from Croatia. Adults usually found around small muddy springs and slowly flowing streams with rich organic muds.

**Figure 16.**
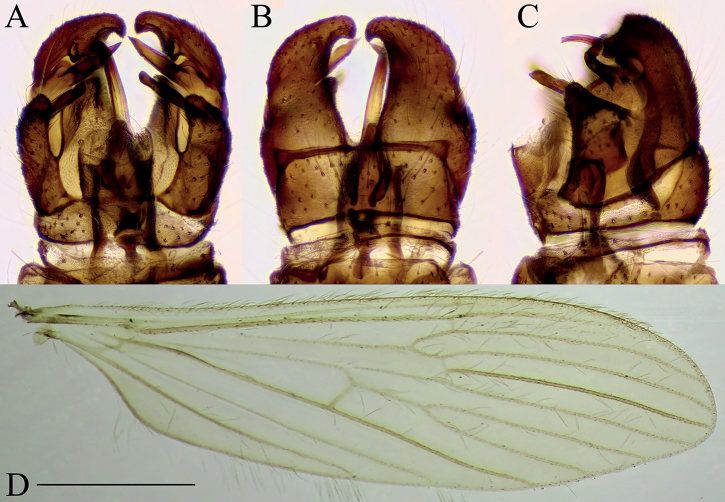
Molophilus (Molophilus) pullus, specimen: Croatia, Zagreb, stream Jelenovac (CKLP) **A–C** male terminalia **A** dorsal view **B** ventral view **C** lateral view **D** wing. Scale bar: 1 mm (**D**).

##### ﻿45. Neolimonia dumetorum

Taxon classificationAnimaliaDipteraLimoniidae

(Meigen, 1804)

C6761B79-559D-5974-9C4C-98713187D0AE

###### Material examined.

**Croatia** • 2 females; Ličko-Senjska county, near Baške Oštarije, Velebit NP; 44.545°N, 15.152°E; alt. 980 m; 30 July 2021; sweep net; leg. M.C. d’Oliveira; PCMCO. **Slovenia** • 1 male; Gorenjska, near Spominski Park; 46.336°N, 14.573°E; alt. 730 m; leg. M.C. de Haas; PCMCO.

###### Comments.

A common species.

##### ﻿46. Orimarga (Orimarga) juvenilis

Taxon classificationAnimaliaDipteraLimoniidae

(Zetterstedt, 1851)

C23E3A51-465C-59F6-8EC9-75F869CD1DB6

###### Material examined.

**Slovenia** • 1 male, 1 female; Gorenjska, Kranjska Gora, Gozd Maturljek, in small woodland near river Sava; in small woodland on the banks of river Sava; 46.483°N, 13.838°E; alt. 740 m; 20 July 2019; light trap; leg. M.C. d’Oliveira; PCMCO.

###### Comments.

A rare species, known from few European countries. *Orimarga* species are associated with hygropetric calcareous habitats. First record from Slovenia.

##### ﻿47. Orimarga (Orimarga) virgo

Taxon classificationAnimaliaDipteraLimoniidae

(Zetterstedt, 1851)

8B83507B-F5EE-52C0-B230-EDA494DA67D5

###### Material examined.

**Slovenia** • 1 male; Vintgar gorge; 46.393333°N, 14.086056°E; alt. 600 m; 1 July 2022; leg C.Quindroit; PCCQ.

###### Comments.

Another rare *Orimarga* species.

##### ﻿48. Ormosia (Ormosia) albitibia

Taxon classificationAnimaliaDipteraLimoniidae

Edwards, 1921

D63B825A-0D2D-5D55-9F7C-9255A6E2611E

###### Material examined.

**Slovenia** • 1 male; Krma Valley; 46.370556°N, 13.88916°E; in low alder tree area, in a rockslide; alt. 900 m; 6 July 2022; leg. C. Quindroit; PCCQ.

###### Comments.

A rare species, usually found around mountain streams in Central Europe. First record from Slovenia.

##### ﻿49. Ormosia (Ormosia) lineata

Taxon classificationAnimaliaDipteraLimoniidae

(Meigen, 1804)

3C42E41D-309A-54C4-9B11-2B77A1712F6B

Fig. 17

###### Material examined.

**Croatia** • 1 male, 1 female; Zagreb, Stream Jelenovac; 45.82306°N, 15.95235°E; alt. 170 m; 10 April 2020; leg. M. Ivković; CKLP.

###### Comments.

A common early spring species usually found around muddy, sandy springs and smaller streams. First record from Croatia.

**Figure 17.**
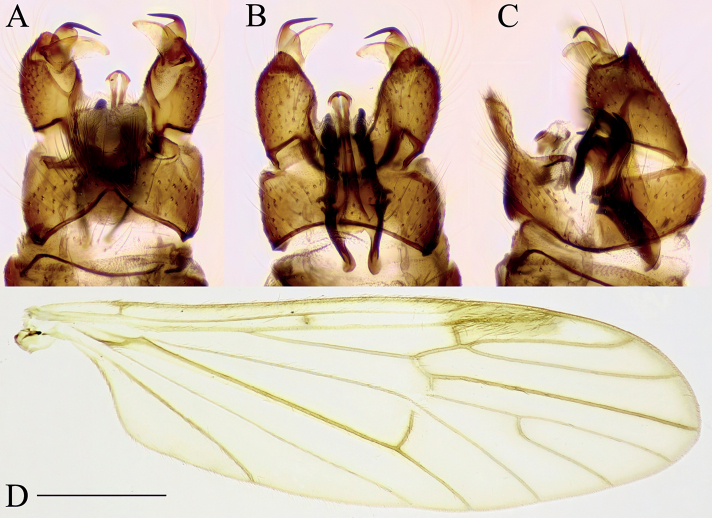
Ormosia (Ormosia) lineata, Specimen: Zagreb, stream Jelenovac (CKLP) **A–C** male terminalia **A** dorsal view **B** ventral view **C** lateral view **D** wing. Scale bar: 1 mm (**D**).

##### ﻿50. Paradelphomyia fuscula

Taxon classificationAnimaliaDipteraLimoniidae

(Loew, 1873)

B08B7BC8-DCBF-5F42-85F9-DBA3D0538E62

###### Material examined.

**Croatia** • 1 male; Ličko-Senjska county, Plitvički Ljeskovac, Tufa barrier Labudovac, Plitvice Lakes; 44.87138°N, 15.59972°E; alt. 630 m; 30 June 2021; emergence trap, P7; leg. M. Ivković; CKLP.

###### Comments.

A species associated with various aquatic and semi-aquatic habitats. First record from Croatia.

##### ﻿51. Paradelphomyia senilis

Taxon classificationAnimaliaDipteraLimoniidae

(Haliday, 1833)

C5B0B454-C5CC-5509-8063-ECF100F43C8A

###### Material examined.

**Croatia** • 1 male; Ličko-Senjska county, Plitvički Ljeskovac, Tufa barrier Labudovac, Plitvice Lakes; 44.87138°N, 15.59972°E; alt. 630 m; 30 June 2015; emergence trap, P6; leg. M. Ivković; CKLP.

###### Comments.

A widespread species in Europe.

##### ﻿52. Prionolabis hospes

Taxon classificationAnimaliaDipteraLimoniidae

(Egger, 1863)

E240EE32-059B-51E6-82E2-7E67FBB9BC3E

###### Material examined.

**Slovenia** • 1 male; Vintgar gorge; 46.393333°N, 14.086056°E; alt. 600 m; 1 July 2022; leg C. Quindroit; PCCQ.

###### Comments.

A common species associated with deciduous forests.

##### ﻿53. Symplecta (Psiloconopa) sticticastictica

Taxon classificationAnimaliaDipteraLimoniidae

(Meigen, 1818)

353A5380-63FB-5DDB-A38D-3D909CFE284E

###### Material examined.

**Albania** • 1 female; Shkodër, Lëpushë; 42.5291°N, 19.72654°E; alt. 1290 m; 3 August 2021; leg. A. de Ketelaere; PCMCO.

###### Comments.

A common and widespread species, occurs in different semi-aquatic habitats, also tolerant of salty soils.

##### ﻿54. Thaumastoptera (Thaumastoptera) calceata

Taxon classificationAnimaliaDipteraLimoniidae

Mik, 1866

8819F80D-EAC3-53F8-BBBD-0DB2EFA3EED5

###### Material examined.

**Bosnia and Herzegovina** • 1 male; Ulog, Ulog on Neretva River; 43.42414°N, 18.30837°E; alt. 640 m; 29 June 2022; leg. W. Graf; CKLP. **Croatia** • 1 male; Ličko-Senjska county, Rastovača, Tufa barrier Kozjak-Milanovac, Plitvice Lakes; 44.89416°N, 15.60888°E; alt. 545 m; 30 June 2015; emergence trap, P5 • 1 male; same locality; 30 June 2019; emergence trap, P1; leg. M. Ivković; CKLP.

###### Comments.

A relative rare and tiny species, associated with calcareous rivers and streams usually with sandy banks. First records from Croatia and Bosnia and Herzegovina.

#### ﻿Pediciidae

##### ﻿55. Dicranota (Paradicranota) subtilis

Taxon classificationAnimaliaDiptera﻿Pediciidae

Loew, 1871

5DF37343-BE18-568B-BEFA-B4F7A827CFAF

Fig. 18

###### Material examined.

**Croatia** • 1 male; Ličko-Senjska county, Končarev Kraj, Spring of Bijela rijeka, Plitvice Lakes; 44.83472°N, 15.56194°E; alt. 720 m; 28 April 2017; emergence trap, P5 • 1 male; same locality; 29 October 2020; emergence trap, P5 • 1 male; same locality; 28 October 2021; emergence trap, P1; leg. M. Ivković; CKLP.

###### Comments.

One of the most common *Dicranota* species in Europe, adults frequently collected around different types of small streams and mountain rivers. First records from Croatia.

**Figure 18.**
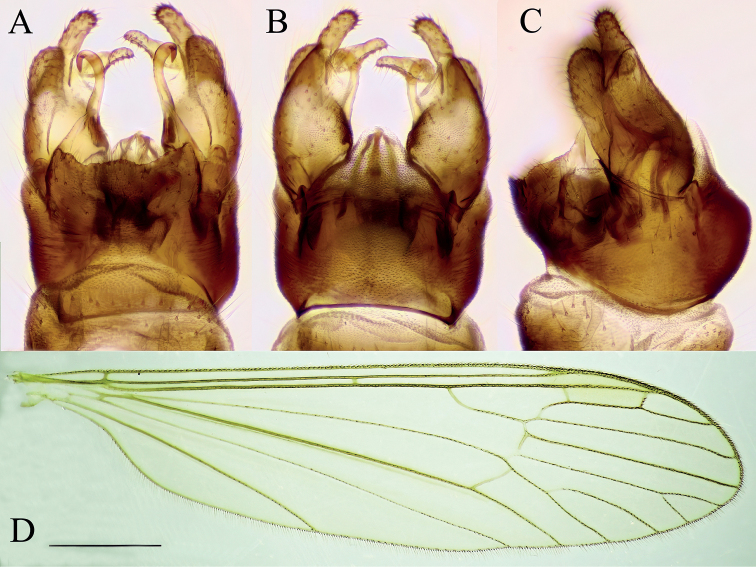
Dicranota (Paradicranota) subtilis, Specimen: Croatia, spring of Bijela rijeka, Plitvice Lakes (CKLP) **A–C** male terminalia **A** dorsal view **B** ventral view **C** lateral view **D** wing. Scale bar: 1 mm (**D**).

##### ﻿56. Pedicia (Amalopis) occulta

Taxon classificationAnimaliaDiptera﻿Pediciidae

(Meigen, 1830)

BF6B15DF-ABA5-52CD-B890-1510499D7D55

###### Material examined.

**Croatia** • 1 female; Ličko-Senjska county, Končarev Kraj, Spring of Bijela rijeka, Plitvice Lakes; 44.83472°N, 15.56194°E; alt. 720 m; 31 March 2014; emergence trap, P5 • 2 males, 2 females; same locality; 30 April 2014; emergence trap, P5 • 2 females; same locality; 30 April 2014; emergence trap, P6 • 1 female; same locality; 29 May 2014; emergence trap, P4 • 1 male; same locality; 30 June 2014; emergence trap, P2 • 1 male; same locality; 30 September 2014; emergence trap, P2 • 1 male; same locality; 29 April 2015; emergence trap, P4 • 1 female; same locality; 29 April 2015; emergence trap, P5 • 1 female; same locality; 30 September 2015; emergence trap, P3 • 1 male; same locality; 31 March 2016; emergence trap, P3 • 1 female; same locality; 31 May 2016; emergence trap, P3 • 1 male; same locality; 31 May 2016; emergence trap, P6 • 1 female; same locality; 31 August 2016; emergence trap, P3 • 1 male, 1 female; same locality; 31 October 2016; emergence trap, P3 • 1 male; same locality; 29 March 2017; emergence trap, P4 • 2 females; same locality; 28 April 2017; emergence trap, P4 • 1 male; same locality; 28 June 2017; emergence trap, P4 • 1 male; same locality; 27 July 2017; emergence trap, P4 • 1 male; same locality; 27 July 2017; emergence trap, P3 • 1 male; same locality; 30 August 2017; emergence trap, P4 • 1 female; same locality; 28 October 2017; emergence trap, P5 • 1 female; same locality; 28 October 2017; emergence trap, P6 • 1 female; same locality; 29 November 2017; emergence trap, P3 • 1 female; same locality; 29 November 2017; emergence trap, P6 • 1 female; same locality; 30 March 2018; emergence trap, P6 • 1 male, 1 female; same locality; 30 April 2018; emergence trap, P6 • 1 male; same locality; 29 June 2018; emergence trap, P5 • 1 female; same locality; 29 June 2018; emergence trap, P6 • 1 male; same locality; 26 July 2018; emergence trap, P6 • 1 female; same locality; 28 May 2019; emergence trap, P5 • 1 female; same locality; 27 July 2019; emergence trap, P4 • 1 male; same locality; 30 April 2020; emergence trap, P5 • 1 female; same locality; 28 May 2020; emergence trap, P6 • 3 males, 3 females; same locality; 29 October 2020; emergence trap, P3 • 1 male; same locality; 28 February 2021; emergence trap, P3 • 1 male; same locality; 30 March 2021; emergence trap, P3 • 1 female; same locality; 29 April 2021; emergence trap, P3 • 1 male; same locality; 29 April 2021; emergence trap, P2 • 1 female; same locality; 29 April 2021; emergence trap, P5 • 1 male; same locality; 29 May 2021; emergence trap, P3 • 1 male, 1 female; same locality; 29 July 2021; emergence trap, P3 • 1 male; same locality; 29 July 2021; emergence trap, P6 • 1 female; same locality; 31 August 2021; emergence trap, P3 • 1 female; same locality; 31 August 2021; emergence trap, P6 • 1 male; same locality; 28 October 2021; emergence trap, P6 • 1 male; same locality; 28 October 2021; emergence trap, P3 • 1 female; same locality; 29 November 2021; emergence trap, P3; leg. M. Ivković; CKLP • 1 male, 1 female; Ličko-Senjska county, Plitvica Selo, Stream Plitvica, Plitvice Lakes; 44.90222°N, 15.6075°E; alt. 555 m; 30 September 2008; emergence trap, P3 • 5 males, 2 females; same locality; 30 September 2008; emergence trap, P2 • 1 female; same locality; 30 October 2008; emergence trap, P2; leg. M. Ivković; UZC.

###### Comments.

A widely distributed species, one of the most common and abundant species occurring together with *Dicranomyiadidyma* in samples collected with emergence traps at Plitvice Lakes.

##### ﻿57. Pedicia (Crunobia) littoralis

Taxon classificationAnimaliaDiptera﻿Pediciidae

(Meigen, 1804)

44067898-C159-5FFF-B6EA-AEFF19A3B122

###### Material examined.

**Slovenia** • 1 male; Kobarid, Kozjak waterfall; 46.259139°N, 13.590056°E; alt. 360 m; 9 July 2022; leg C. Quindroit; PCCQ.

###### Comments.

A widely distributed *Crunobia* species, can be found around small, cold-water streams. The males swarm above littoral vegetation.

##### ﻿58. Pedicia (Crunobia) straminea

Taxon classificationAnimaliaDiptera﻿Pediciidae

(Meigen, 1838)

A437DABE-C487-5F63-92C3-C38D623FD6C7

###### Material examined.

**Slovenia** • 1 male; Vintgar gorge; 46.393333°N, 14.086056°E; alt. 600 m; 1 July 2022; leg C. Quindroit; PCCQ.

###### Comments.

Another common and widely distributed *Crunobia* species, can be found throughout a wider range of habitats than *P.littoralis*, including springs and small, cold-water rivers.

##### ﻿59. Pedicia (Pedicia) rivosa

Taxon classificationAnimaliaDiptera﻿Pediciidae

(Linnaeus, 1758)

AF647A68-4291-56F7-80C2-0544B01D0C61

###### Material examined.

**Croatia** • 1 female; Ličko-Senjska county, Končarev Kraj, Spring of Bijela rijeka, Plitvice Lakes; 44.83472°N, 15.56194°E; alt. 720 m; 29 May 2018; emergence trap, P5 • 1 female; same locality; 28 June 2020; emergence trap, P1; leg. M. Ivković; CKLP.

###### Comments.

A relative common and easily recognizable species. Larvae are aquatic and the species can be found around springs and streams, from lowlands to high mountains. Appears to be a very rare species in southern part of Europe and found only in humid mountains in the Western Balkans. Here we present the first records from Croatia.

##### ﻿60. Tricyphona (Tricyphona) immaculata

Taxon classificationAnimaliaDiptera﻿Pediciidae

(Meigen, 1804)

F46AD51F-6E0F-5BB9-ABE8-7451FD14CF7D

###### Material examined.

**Croatia** • 1 male; Ličko-Senjska county, Končarev Kraj, Spring of Bijela rijeka, Plitvice Lakes; 44.83472°N, 15.56194°E; alt. 720 m; 03 January 2019; emergence trap, P4 • 2 males; same locality; 29 October 2020; emergence trap, P5 • 1 male; same locality; 28 October 2021; emergence trap, P1; leg. M. Ivković; CKLP • 1 male; Ličko-Senjska county, Plitvički Ljeskovac, Tufa barrier Labudovac, Plitvice Lakes; 44.87138°N, 15.59972°E; alt. 630 m; 29 October 2020; emergence trap, P3 • 1 male; same locality; 29 September 2021; emergence trap, P6; leg. M. Ivković; UZC.

###### Comments.

A relative common and wide distributed species, associated with different types of aquatic and semi-aquatic habitats.

#### ﻿Tipulidae

##### ﻿61. Ctenophora (Cnemoncosis) ornata

Taxon classificationAnimaliaDiptera﻿Tipulidae

Meigen & Wiedemann, 1818

2CE3A38B-482F-517F-BED4-DC7019D97B2A

###### Material examined.

**Croatia** • 1 female; Zadarska county, Benkovac, Pristeg; 43.96214°N, 15.62876°E; alt. 170 m; 01 April 2021; leg. T. Dražina; CKLP.

###### Comments.

Conspicuous species associated with old decaying standing wood.

##### ﻿62. Nephrotoma appendiculata appendiculata

Taxon classificationAnimaliaDiptera﻿Tipulidae

(Pierre, 1919)

320D7345-09E2-5639-8583-053BC2DF7FAB

###### Material examined.

**Croatia** • 1 male, 1 female; Zagrebačka county, Kuče, Chanel Sava Odra; 45.6725°N, 16.135278°E; alt. 98 m; 14 April 2011; leg. M. Ivković; CKLP.

###### Comments.

A very common species.

##### ﻿63. Nephrotoma cornicina cornicina

Taxon classificationAnimaliaDiptera﻿Tipulidae

(Linnaeus, 1758)

2B717FCD-7014-5A17-8348-14FCF7D141A2

###### Material examined.

**Slovenia** • 1 male; Češnjica; 46.296667°N, 13.948917°E; alt. 600 m; 1 July 2022; leg. C. Quindroit; PCCQ.

###### Comments.

A very common species.

##### ﻿64. Nephrotoma flavescens

Taxon classificationAnimaliaDiptera﻿Tipulidae

(Linnaeus, 1758)

7EB26B6F-B871-566D-8190-E41ED5EE2852

###### Material examined.

**Bosnia and Herzegovina** • 1 male; Ulog, Ulog on Neretva River; 43.42414°N, 18.30837°E; alt. 640 m; 29 June 2022; leg. W. Graf; CKLP.

###### Comments.

A common species.

##### ﻿65. Nephrotoma lamellata lamellata

Taxon classificationAnimaliaDiptera﻿Tipulidae

(Riedel, 1910)

ED1A6621-9EA5-5E41-88C8-9034552D268F

###### Material examined.

**Slovenia** • 1 male; Češnjica; 46.296667°N, 13.948917°E; alt. 600 m; 1 July 2022; leg. C. Quindroit; PCCQ.

###### Comments.

A less common *Nephrotoma* species.

##### ﻿66. Nephrotoma quadrifaria quadrifaria

Taxon classificationAnimaliaDiptera﻿Tipulidae

(Meigen, 1804)

0F61EBAA-3755-5D97-9768-C5E831B6EAA2

###### Material examined.

**Bosnia and Herzegovina** • 1 female; Cerova, Cerova on Neretva; 43.37887°N, 18.35621°E; alt. 695 m; 30 June 2022; leg. M. Ivković; CKLP.

###### Comments.

A common species.

##### ﻿67. Tipula (Acutipula) balcanica

Taxon classificationAnimaliaDiptera﻿Tipulidae

Vermoolen, 1983

67B826A5-1597-5816-9F46-6D6903BD59FE

###### Material examined.

**Croatia** • 1 male; Ličko-Senjska county, Plitvički Ljeskovac, Tufa barrier Labudovac, Plitvice Lakes; 44.87138°N, 15.59972°E; alt. 630 m; 30 June 2019; emergence trap, P6; leg. M. Ivković; CKLP.

###### Comments.

A relatively common and conspicuous species in the Balkans and some neighboring countries.

##### ﻿68. Tipula (Lunatipula) fascipennis

Taxon classificationAnimaliaDiptera﻿Tipulidae

Meigen, 1818

13CA5487-373D-5C37-9027-A6A9F98F147E

###### Material examined.

**Bosnia and Herzegovina** • 1 male; Ulog, Ulog on Neretva River; 43.42414°N, 18.30837°E; alt. 640 m; 29 June 2022; leg. W. Graf; CKLP.

###### Comments.

A common species.

##### ﻿69. Tipula (Lunatipula) helvola

Taxon classificationAnimaliaDiptera﻿Tipulidae

Loew, 1873

67BCAF7A-0972-5B9A-BDBE-C7EA557A5950

###### Material examined.

**Slovenia** • 1 male; Ljubljana, Castle; 46.048°N, 14.509778°E; alt. 340 m; 30 June 2022; leg. C. Quindroit; PCCQ.

###### Comments.

A common species.

##### ﻿70. Tipula (Lunatipula) truncata

Taxon classificationAnimaliaDiptera﻿Tipulidae

Loew, 1873

39D692EB-85E9-561C-8E82-D9A2B26A1F8E

###### Material examined.

**Bosnia and Herzegovina** • 1 male, 2 females; Ulog, Ulog on Neretva River; 43.42414°N, 18.30837°E; alt. 640 m; 29 June 2022; leg. W. Graf; CKLP.

###### Comments.

A common species in central and southeastern Europe, prefers drier habitats.

##### ﻿71. Tipula (Pterelachisus) glacialis

Taxon classificationAnimaliaDiptera﻿Tipulidae

(Pokorny, 1887)

BA117918-B122-550F-B03A-CB81348D89BA

###### Material examined.

**Slovenia** • 1 male; Krnica; 46.37333°N, 13.862528°E; alt. 2000 m; 6 July 2022; leg. C. Quindroit; PCCQ • 2 males; Dom Planika; 46.371417°N, 13.846417°E; alt. 2400 m; 6 July 2022; leg. C. Quindroit; PCCQ • 1 male; Rudno Polje, Sreniski preval; 46.360889°N, 13.894472°E; alt. 1900 m; 3 July 2022; leg. C. Quindroit; PCCQ.

###### Comments.

A rare alpine species, known only from the Alps, Dinaric Alps, and Rila-Rhodope massif.

##### ﻿72. Tipula (Savtshenkia) benesignata

Taxon classificationAnimaliaDiptera﻿Tipulidae

Mannheims, 1954

4CE2FF97-933F-5475-A514-D8BC6C56E59D

###### Material examined.

**Croatia** • 1 male; Ličko-Senjska county, Rastovača, Tufa barrier Kozjak-Milanovac, Plitvice Lakes; 44.89416°N, 15.60888°E; alt. 545 m; 28 September 2020; emergence trap, P1; leg. M. Ivković; CKLP

###### Comments.

A relative common autumnal species.

##### ﻿73. Tipula (Savtshenkia) cheethami

Taxon classificationAnimaliaDiptera﻿Tipulidae

Edwards, 1924

1AD72348-FD40-5091-9335-EDE61169575F

###### Material examined.

**Croatia** • 2 males; Ličko-Senjska county, Končarev Kraj, Spring of Bijela rijeka, Plitvice Lakes; 44.83472°N, 15.56194°E; alt. 720 m; 29 May 2018; emergence trap, P5 • 1 male; same locality; 29 May 2018; emergence trap, P5 • 1 male; same locality; 29 May 2021; emergence trap, P6; leg. M. Ivković; CKLP • 1 male; Ličko-Senjska county, Plitvički Ljeskovac, Spring of Crna rijeka, Plitvice Lakes; 44.83714°N, 15.60752°E; alt. 680 m; 29 May 2021; emergence trap, P6; leg. M. Ivković; UZC.

###### Comments.

A rare, late spring species, associated with wet, mossy habitats along rivers and streams. First records from Croatia.

##### ﻿74. Tipula (Savtshenkia) rufinarufina

Taxon classificationAnimaliaDiptera﻿Tipulidae

Meigen, 1818

722999F3-44B3-51AC-89D7-F0ADB5EAE23E

###### Material examined.

**Croatia** • 1 male; Ličko-Senjska county, Končarev Kraj, Spring of Bijela rijeka, Plitvice Lakes; 44.83472°N, 15.56194°E; alt. 720 m; 31 October 2016; emergence trap, P6 • 2 males; same locality; 30 September 2019; emergence trap, P5 • 3 males, 1 female; same locality; 29 September 2021; emergence trap, P6; leg. M. Ivković; CKLP • 1 male; Ličko-Senjska county, Rastovača, Tufa barrier Kozjak-Milanovac, Plitvice Lakes; 44.89416°N, 15.60888°E; alt. 545 m; 28 September 2020; emergence trap, P3 • 1 female; same locality; 29 October 2020; emergence trap, P5; leg. M. Ivković; UZC.

###### Comments.

A common and widespread species, known from different habitats.

##### ﻿75. Tipula (Vestiplex) hortorum

Taxon classificationAnimaliaDiptera﻿Tipulidae

Linnaeus, 1758

C42B687D-C57F-5153-B124-A85F491D6BC4

###### Material examined.

**Croatia** • 1 female; Ličko-Senjska county, Končarev Kraj, Spring of Bijela rijeka, Plitvice Lakes; 44.83472°N, 15.56194°E; alt. 720 m; 28 June 2017; emergence trap, P5; leg. M. Ivković; CKLP • 1 male; Grad Zagreb County, Zagreb, Stream Jelenovac; 45.82306°N, 15.95235°E; alt. 170 m; 10 April 2020; leg. M. Ivković; CKLP.

###### Comments.

A common species, usually found in wet forest habitats.

##### ﻿76. Tipula (Vestiplex) excisaexcisa

Taxon classificationAnimaliaDiptera﻿Tipulidae

Schummel, 1833

45BE6656-C9DD-54B6-B12A-AAE39C97FA6A

###### Material examined.

**Slovenia** • 1 male; Krnica; 46.373333°N, 13.862528°E; alt. 2000 m; 6 July 2022; leg. C. Quindroit; PCCQ.

###### Comments.

A mountain alpine species.

##### ﻿77. Tipula (Yamatotipula) lateralis

Taxon classificationAnimaliaDiptera﻿Tipulidae

Meigen, 1804

2C717A76-4468-5CDF-9639-1CE0ECCB0B3F

###### Material examined.

**Bosnia and Herzegovina** • 1 male; Ulog, Ulog on Neretva River; 43.42414°N, 18.30837°E; alt. 640 m; 29 June 2022; leg. W. Graf; CKLP.

###### Comments.

A very common aquatic/semi-aquatic species.

## ﻿Discussion

As many other publications have previously demonstrated, the biodiversity of the Balkans remains insufficiently investigated, and the local fauna consists of far more species than what is currently reported (e.g., Ivković et al. 2013; Kvifte et al. 2013; Pont and Ivković 2013; Ivković and Plant 2015; Ivković and Pont 2015; Ivković et al. 2016; Kvifte and Ivković 2018; Keresztes et al. 2018b). Despite historical and more recent publications (Bilalli et al. 2021; de Jong et al. 2021; Kolcsár et al. 2015a, b, 2017a, b, 2018a, b, 2023; Keresztes et al. 2018a, b; Starý 2012) the cranefly fauna of the Western Balkans is still poorly known. In this study we report two species for the first time from Albania, eight from Bosnia and Herzegovina, twelve from Croatia, and seven from Slovenia. Most of the newly reported species are common and widely distributed in Europe, highlighting how understudied the local fauna is. However, some rare and poorly known species such as *Antochaalpigena*, *Ellipteroideslimbatus*, *Idiocerajucunda*, *I.lackschewitzi*, and *Orimargajuvenilis* etc. were also collected, primarily around natural streams and rivers. Furthermore, a new species, *Baeouraneretvaensis* sp. nov. is described. *Baeoura* is considered as a rare group in the Western Palaearctic, with a distribution mainly restricted to the Mediterranean region (Fig. 5). Besides the original species descriptions (Kuntze 1914; Mendl and Tjeder 1974, 1976; Starý 1981; Krzemiński and Starý 1984; Mendl 1985, 1986; Driauach and Belqat 2015; Hancock 2020) only few additional faunistic records are known from the Western Palearctic (Erhan-Dincă 1984; Simova-Tosic 1992; Starý and Oosterbroek 1996; Koç 2004; Ujvárosi 2005; Starý 2014; Oosterbroek et al. 2020; Kolcsár et al. 2021).

The biology and ecology of *Baeoura* species are poorly known or even unknown. Larvae and pupae of the South African species *B.claripennis* (Alexander, 1921) were found on the surface of smaller boulders, ~ 30 cm deep in a swiftly flowing stream and the imago was collected from vegetation along the stream (Wood 1952). Adults of *B.malickyi*, *B.schachti* Mendl, 1986, and *B.armata* Mendl, 1985 were collected around small streams with clear water (Mendl and Tjeder 1976; Mendl 1985, 1986; Kolcsár et al. 2021). *Baeourarotherayi* and *B.szadziewskii* Krzemiński & Starý, 1984 were also collected along streams (Krzemiński and Starý 1984; Hancock 2020). Driauach and Belqat (2015) collected *B.ebenina* Starý, 1981 and *B.staryi* along partly dried out rivers, with minimal water flow in Morocco. Adults of *B.alexanderi* Mendl & Tjeder, 1974 in Crete and *B.longefiligera* Mendl, 1986 in Spain were found in dry valleys of very small streams (Mendl and Tjeder 1974; Mendl 1986). It is assumed that larvae of Western Palaearctic species are also aquatic or semi-aquatic (Mendl and Tjeder 1976; Erhan-Dincă 1984). Adults of *B.neretvaensis* sp. nov. were collected around fast flowing gravelly/rocky streams and rivers (Fig. 4). *Baeouramalickyi* and *B.neretvaensis* sp. nov. were collected from the same habitat at the same time along Sava River in Slovenia (Fig. 5, see also Kolcsár et al. 2021). Based on the above-mentioned observations, the European *Baeoura* species are rheobiont, larvae most probably occurring in fast flowing streams and rivers. Some species seem to be adapted to fluctuating water conditions, tolerating the partly or fully drying up of streams and rivers during the dry periods in the Mediterranean area. The adults migrate farther from the larval habitats along valleys. Based on literature data and our observations, *Baeoura* species are attracted by light sources and surveillance traps could be effective methods to collect these small-sized and poorly known species.

## ﻿Conclusions

At this time of intensified climate change, that causes streams and rivers to dry up, and the general increasing anthropogenic influences on freshwater habitats, we stipulate that it is of utmost importance to protect the streams and rivers as much as we possibly can. The Western Balkans is a part of Europe with still relatively little human impact and therefore our efforts on exploring and studying this area need to be much more than they are at present. Only by increasing our knowledge of the diversity of this area can we help to protect it more thoroughly. It truly deserves to be protected, so it can remain the Blue Heart of Europe.

## Supplementary Material

XML Treatment for Baeoura

XML Treatment for Baeoura neretvaensis

XML Treatment for Antocha (Antocha) vitripennis

XML Treatment for Antocha (Orimargula) alpigena

XML Treatment for Atypophthalmus (Microlimonia) machidai

XML Treatment for Austrolimnophila (Austrolimnophila) ochracea

XML Treatment for Dicranomyia (Dicranomyia) chorea

XML Treatment for Dicranomyia (Dicranomyia) conchifera

XML Treatment for Dicranomyia (Dicranomyia) didyma

XML Treatment for Dicranomyia (Dicranomyia) imbecilla

XML Treatment for Dicranomyia (Dicranomyia) mitis

XML Treatment for Dicranomyia (Dicranomyia) quadra

XML Treatment for Dicranomyia (Melanolimonia) morio

XML Treatment for Dicranomyia (Melanolimonia) stylifera

XML Treatment for Dicranomyia (Numantia) fusca

XML Treatment for Dicranomyia (Sivalimnobia) aquosa

XML Treatment for Dicranophragma (Brachylimnophila) nemorale

XML Treatment for Dicranoptycha fuscescens

XML Treatment for Elliptera omissa

XML Treatment for Ellipteroides (Ellipteroides) lateralis

XML Treatment for Ellipteroides (Protogonomyia) alboscutellatus

XML Treatment for Ellipteroides (Protogonomyia) limbatus

XML Treatment for Eloeophila miliaria

XML Treatment for Erioptera (Erioptera) lutealutea

XML Treatment for Gonomyia (Prolipophleps) abbreviata

XML Treatment for Helius (Helius) pallirostris

XML Treatment for Hexatoma (Eriocera) chirothecata

XML Treatment for Hexatoma (Hexatoma) bicolor

XML Treatment for Idiocera (Euptilostena) jucunda

XML Treatment for Idiocera (Idiocera) lackschewitzi

XML Treatment for Ilisia maculata

XML Treatment for Limonia nubeculosa

XML Treatment for Limonia sylvicola

XML Treatment for Limonia taurica

XML Treatment for Limonia phragmitidis

XML Treatment for Lipsothrix nobilis

XML Treatment for Lipsothrix remota

XML Treatment for Molophilus (Molophilus) aduncus

XML Treatment for Molophilus (Molophilus) appendiculatus

XML Treatment for Molophilus (Molophilus) bifidus

XML Treatment for Molophilus (Molophilus) corniger

XML Treatment for Molophilus (Molophilus) crassipygus

XML Treatment for Molophilus (Molophilus) lackschewitzianus

XML Treatment for Molophilus (Molophilus) medius

XML Treatment for Molophilus (Molophilus) propinquus

XML Treatment for Molophilus (Molophilus) pullus

XML Treatment for Neolimonia dumetorum

XML Treatment for Orimarga (Orimarga) juvenilis

XML Treatment for Orimarga (Orimarga) virgo

XML Treatment for Ormosia (Ormosia) albitibia

XML Treatment for Ormosia (Ormosia) lineata

XML Treatment for Paradelphomyia fuscula

XML Treatment for Paradelphomyia senilis

XML Treatment for Prionolabis hospes

XML Treatment for Symplecta (Psiloconopa) sticticastictica

XML Treatment for Thaumastoptera (Thaumastoptera) calceata

XML Treatment for Dicranota (Paradicranota) subtilis

XML Treatment for Pedicia (Amalopis) occulta

XML Treatment for Pedicia (Crunobia) littoralis

XML Treatment for Pedicia (Crunobia) straminea

XML Treatment for Pedicia (Pedicia) rivosa

XML Treatment for Tricyphona (Tricyphona) immaculata

XML Treatment for Ctenophora (Cnemoncosis) ornata

XML Treatment for Nephrotoma appendiculata appendiculata

XML Treatment for Nephrotoma cornicina cornicina

XML Treatment for Nephrotoma flavescens

XML Treatment for Nephrotoma lamellata lamellata

XML Treatment for Nephrotoma quadrifaria quadrifaria

XML Treatment for Tipula (Acutipula) balcanica

XML Treatment for Tipula (Lunatipula) fascipennis

XML Treatment for Tipula (Lunatipula) helvola

XML Treatment for Tipula (Lunatipula) truncata

XML Treatment for Tipula (Pterelachisus) glacialis

XML Treatment for Tipula (Savtshenkia) benesignata

XML Treatment for Tipula (Savtshenkia) cheethami

XML Treatment for Tipula (Savtshenkia) rufinarufina

XML Treatment for Tipula (Vestiplex) hortorum

XML Treatment for Tipula (Vestiplex) excisaexcisa

XML Treatment for Tipula (Yamatotipula) lateralis
